# Unveiling Consumer Preferences and Intentions for Cocreated Features of a Combined Diet and Physical Activity App: Cross-Sectional Study in 4 European Countries

**DOI:** 10.2196/44993

**Published:** 2023-12-11

**Authors:** Bahram Mahmoodi Kahriz, Sarah Snuggs, Anumeha Sah, Sophie Clot, Daniel Lamport, Joseph Forrest, Agnes Helme-Guizon, Marie-Claire Wilhelm, Cindy Caldara, Camille Valentine Anin, Julia Vogt

**Affiliations:** 1 Henley Business School University of Reading Reading United Kingdom; 2 School of Psychology and Clinical Language Sciences University of Reading Reading United Kingdom; 3 Department of Economics University of Reading Reading United Kingdom; 4 Grenoble Alpes University, Grenoble INP, CERAG Grenoble France

**Keywords:** mobile apps, healthy eating and physical activity, attitude, BMI and self-efficacy

## Abstract

**Background:**

Numerous mobile health apps are marketed globally, and these have specific features including physical activity tracking, motivational feedback, and recipe provision. It is important to understand which features individuals prefer and whether these preferences differ between consumer groups.

**Objective:**

In this study, we aimed to identify consumers’ most preferred features and rewards for a mobile app that targets healthy eating and physical activity and to reduce the number of individual mobile health app features to a smaller number of key categories as perceived by consumers. In addition, we investigated the impact of differences in consumers’ BMI and self-efficacy on their intention to use and willingness to pay for such an app. Finally, we identified the characteristics of different target groups of consumers and their responses toward app features via cluster analysis.

**Methods:**

A total of 212 participants from France, Italy, the United Kingdom, and Germany were recruited via the web to answer questions about app features, motivation, self-efficacy, demographics, and geographic factors. It is important to note that our study included an evenly distributed sample of people in the age range of 23 to 50 years (23-35 and 35-50 years). The app features in question were generated from a 14-day cocreation session by a group of consumers from the United Kingdom and the Republic of Ireland.

**Results:**

“Home work out suggestions,” “exercise tips,” and “progress charts” were the most preferred app features, whereas “gift vouchers” and “shopping discounts” were the most preferred rewards. “Connections with other communication apps” was the least preferred feature, and “charitable giving” was the least preferred reward. Importantly, consumers’ positive attitude toward the “social support and connectedness and mindfulness” app feature predicted willingness to pay for such an app (β=.229; *P*=.004). Differences in consumers’ health status, motivational factors, and basic demographics moderated these results and consumers’ intention to use and willingness to pay for such an app. Notably, younger and more motivated consumers with more experience and knowledge about health apps indicated more positive attitudes and intentions to use and willingness to pay for this type of app.

**Conclusions:**

This study indicated that consumers tend to prefer app features that are activity based and demonstrate progress. It also suggested a potential role for monetary rewards in promoting healthy lifestyle behaviors. Moreover, the results highlighted the role of consumers’ health status, motivational factors, and socioeconomic status in predicting their app use. These results provide up-to-date, practical, and pragmatic information for the future design and operation of mobile health apps.

## Introduction

### Background

The intention to pursue a healthy lifestyle has increased in recent decades; in high-income countries, individuals are more willing to be physically active, exercise, and eat healthily [1,2]. Physical activity and healthy eating are key priorities for change. When combined, they can help individuals combat the most serious health risk factors such as obesity [3]. Therefore, European researchers and decision makers have acknowledged the underlying connection between physical activity and healthy eating and have combined them in their measurement and intervention programs [3,4]. Technology-based solutions such as mobile apps can motivate and help promote physical activity and healthy eating [5]. Mobile apps refer to software apps “designed to support the functions of performing tasks on smartphones, tablet computers, and other personal mobile devices” [6]. They are emerging as essential tools for health-related behavior change interventions [7-11].

### Health App Features, Rewards, and Cocreation in App Development

Despite this growing popularity, understanding of the most preferred features of health-related apps and differences in such preferences between groups of potential consumers remains limited. Kang [12] established some motivating factors for using mobile apps in general, identifying ease, human connection, and social utility (“getting services such as banking,” “product ordering,” and “getting news and information about weather and travel”) as key factors impacting use intention. With reference to health apps specifically, interface design, multimedia content, customizability, rewards, and social influence have all been suggested as key preferred characteristics [13]. These types of apps can help users track their health throughout the day without the need for professional contact [13-15]. Mobile health apps also allow individuals to connect and share their behavioral and health data with health professionals or peers [14,16]. In their study of Portuguese adolescents, Frontini et al [5] found that the most favored features of mobile health apps for this sample were physical exercise tips and plans, eating tips and nutrition information, physical condition and lifestyle charts, and goal setting. Notifications (alerts sent by an app), advertising, and paid access were among the least favored features of mobile health apps in the study. Nevertheless, few studies have investigated the preferences of other demographic groups in the context of health apps. This is particularly true of apps that focus on both physical activity and healthy eating [17]. The primary aim of this study was to explore adult consumers’ most preferred features for an app designed to support healthy eating and physical activity.

Previous research has revealed that contingency (eg, rewards) is one of the important drivers that can direct individuals’ behaviors [18,19]. Similarly, studies in economics define how rewards can be used as catalysts to change health-related behaviors [20,21]. For instance, Mitchell et al [21] developed the *Carrot Rewards app* to reward Canadians with financial incentives (eg, points could be exchanged for groceries, movies, or air travel) initially for downloading the app and then for completing educational health tests (“microlearning”). They reported that *Carrot Rewards* became the most downloaded health app in Canada and that 60% of consumers indicated very high levels of engagement (eg, completing educational health tests every week with the purpose of enhancing health knowledge and health behaviors). On the basis of evidence showing the importance and variety of rewards (eg, gift vouchers, discounts on shopping, and prizes such as books) in motivating individuals to use mobile apps [22-25], this study also aimed to investigate preferences for reward types in a mobile health app designed to support healthy eating and physical activity.

A limitation of this field is the lack of consideration of consumers’ perspectives when developing mobile health apps [26]. Cocreation with consumers can allow for a more consumer-centric approach to developing app features [27,28]. The cocreation method has been defined as the “joint, collaborative, concurrent, peer-like process of producing new value, both materially and symbolically” [29]. In this method, individuals have a high level of interaction and participation, and they are encouraged to share their ideas and innovate products, services, or solutions to specific problems [30]. Developing app features with consumers can help researchers and app designers analyze the factors driving individuals’ preferences for mobile health apps designed to support behavior change [31]. Studies have highlighted the significance of social support including moral and emotional support from family and friends [32-35] in determining successful behavior change using mobile health apps. They have also emphasized the importance of mindfulness [33,36], goal setting, and support for tracking their progress [37] in overcoming obstacles during behavior changes. Previous research has suggested that providing step-by-step plans and personalized advice [38] and gamification [39,40] can also influence dietary and exercise habits, making them important factors for behavior change. The existing literature on behavior change using mobile health apps, along with the cocreation approach, can provide valuable insights into the features of mobile health apps that support healthy eating and physical activity.

### Consumer Attitudes, BMI, Self-Efficacy, and Health App Use Intention

Al Amin et al [41] found that customers’ favorable attitudes toward mobile food ordering apps were positively related to their intention to continue using them. In particular, they found that customers’ favorable attitudes toward mobile food ordering apps were associated with higher satisfaction and enjoyment from using those apps, which was subsequently related to a higher intention to continue using them. Similarly, Dastjerdi et al [42] found that a technophile attitude, referring to “a person’s openness, interest in and competence with (innovative) technologies,” has a positive impact on both user motives and use intention, thus resulting in a rapid growth in consumer demand. There is also evidence that the relationship between attitudes and health app use is affected by BMI [43-45] and self-efficacy [46]. In particular, previous studies have shown that individuals with higher BMI report a higher intention to use mobile apps to achieve their health behavior goals [47]. In addition, previous studies have indicated that self-efficacy is positively linked to the perceived ease of using mobile health services. Higher self-efficacy is also related to a higher intention to use mobile health services [48]. However, little is known about the potential moderating impact of BMI and self-efficacy on the relationship between app feature attitude and intention to use and willingness to pay for such an app.

### Segmenting Mobile App Consumers and Understanding Health App Users

Furthermore, previous studies have highlighted the importance of identifying the characteristics of mobile app users and have attempted to classify them based on these attributes [49,50]. For instance, Doub et al [50] investigated the characteristics of mobile app consumers in the context of eating behavior and discovered 5 consumer segments based on consumers’ “attitudes towards technology; attitudes towards food and nutrition; use of the internet and mobile devices to explore and socially share food; use of the internet and mobile devices to seek information about food/restaurants; and use of mobile devices and apps to support everyday food-related tasks.” Their study did not show significant differences between the segments across some demographic factors (eg, gender, race, and ethnicity) and socioeconomic status but indicated differences across consumers’ age. For instance, they found that consumers who were aged 18 to 34 years were categorized as “Food-focused App Experimenters” and “App-engaged Food Lovers,” whereas older individuals who were aged 55 to 64 and ≥65 years were categorized as “App- and Food-disengaged” or “App-disengaged Food Utilitarians.” They also found that 33% of consumers were interested in “reading restaurant reviews,” “socially sharing food photos,” and “recipe browsing.” Despite the efforts toward segmentation of mobile app consumers in different contexts such as eating behavior and mobile banking, little is known about the characteristics of different target groups of consumers and their responses toward app features that support both healthy eating and physical activity.

This study aimed to identify consumer preferences for specific features and rewards for mobile apps designed to support both healthy eating and physical activity and to reduce the number of individual mobile health app features to a smaller number of key categories as perceived by consumers. Furthermore, it examined the impact of differences in BMI and self-efficacy on intention to use and willingness to pay for such an app. The final aim of this study was to investigate the characteristics of different target groups of consumers and their responses toward app features via cluster analysis.

## Methods

### Design

In an English-speaking (United Kingdom and Republic of Ireland) web-based community, a cocreative approach was implemented to understand consumers’ attitudes toward the potential features of a healthy eating and exercise app. The cocreation activities and findings regarding general motivators and barriers to health behaviors have been reported in detail elsewhere [51]. The activities took place for 2 weeks, and the final *implementation* phase (2 days) helped participants to think about how to combine healthy eating and physical activity into an app as well as to probe what features they would like to see in such an app. This study focused exclusively on the cocreation activities related to mobile health app development.

Informed by the qualitative data provided by the cocreation community and professional expertise, a questionnaire (see Multimedia Appendix 1 for the full questionnaire) [51] was developed, presenting app ideas and features to consumers in France, Italy, the United Kingdom, and Germany. This study reports this quantitative data.

### Participants

A total of 212 participants were recruited from France, Germany, the United Kingdom, and Italy using the web-based platform, Prolific [52]. Responses from 4 participants were identified as straight-liners (ie, giving identical responses to questions in the measurement using the same response scale) [53] and were excluded from the analysis. One participant who had implausible answers was also excluded. Data from 207 participants were included in the analysis (see Table 1 for demographic details). It is important to mention that our study included an even sample of people in the age range of 23 to 50 years (23-35 and 35-50). However, the participants exhibited diverse age ranges across counties. Among the participants, the following proportions were aged >38 years: 20 (39%) out of 51 in France, 13 (24%) out of 54 in Germany, 15 (29%) out of 51 in the United Kingdom, and 14 (28%) out of 51 in Italy (Table 1). Inclusion criteria required the participants to be aged >18 years and possess the capability to read and understand the language used in their nation. There were no further requirements for inclusion or exclusion in this study.

**Table 1:** Participants’ demographics (n=207).

			France (n=51)		Italy (n=51)		United Kingdom (n=51)		Germany (n=54)
**Gender, n (%)**									
	Man	24 (47)		26 (51)		24 (47)		26 (48)	
	Woman	26 (51)		25 (49)		27 (53)		28 (52)	
	Unknown	1 (2)		0 (0)		0 (0)		0 (0)	
**Age (y)**									
	Whole sample, mean (SD)	35.92 (8.0)		34.49 (6.7)		35.59 (7.1)		34.17 (6.3)	
	A1^a^, n (%)	20 (39)		20 (39)		16 (31)		22 (41)	
	A2^b^, n (%)	11 (22)		17 (33)		20 (39)		19 (35)	
	A3^c^, n (%)	20 (39)		14 (28)		15 (29)		13 (24)	
**Education, n (%)**									
	E1^d^	1 (2)		0 (0)		1 (2)		0 (0)	
	E2^e^	0 (0)		0 (0)		12 (24)		2 (4)	
	E3^f^	2 (4)		22 (43)		9 (18)		1 (2)	
	E4^g^	14 (28)		18 (35)		23 (45)		25 (46)	
	E5^h^	26 (51)		9 (18)		4 (8)		24 (44)	
	E6^i^	4 (8)		2 (4)		1 (2)		1 (2)	
	E7^j^	4 (8)		0 (0)		1 (2)		1 (2)	

^a^A1: <31 years (33rd Percentile).

^b^A2: between 31 and 38 years (66th percentile).

^c^A3: between 38 and 50 years (100th percentile).

^d^E1: <high school.

^e^E2: high school or General Certificate of Secondary Education.

^f^E3: A levels.

^g^E4: bachelor’s degree.

^h^E5: master’s degree.

^i^E6: doctoral degree.

^j^E7: other.

### Ethics Approval

The Ethics Committee at the University of Reading in the United Kingdom granted ethics approval for this research (2020-055-JV).

### Procedure

After reading the participant information and providing consent if they wished to participate, participants were invited to complete the questionnaire. Participants were asked questions regarding the features they would like to see in a mobile health app designed to support health behavior change (see Multimedia Appendix 1 for full questionnaire, including those reported in Snuggs et al [51]).

With the exception of the app feature attitude and demographic questions, all questions were presented as statements on a 7-point agreement Likert scale (1=strongly disagree; 7=strongly agree). The questionnaire was initially developed in English and then translated into French, German and Italian, followed by a *back translation* process to ensure that the meaning was maintained [54,55]. See Tables S1-S7 in Multimedia Appendix 1 for the full questionnaire. Table 2 shows Cronbach α for all scales.

**Table 2:** Information about all the scales.

Scale name				Items, n		Cronbach α		Example item		Rating
App feature attitudes				34		.93		Latest news and trends in eating and exercise		1=has no value at all; 7=extremely valuable
Reward attitudes				5		.69		Gift vouchers		1=strongly disagree; 7=strongly agree
**Self-efficacy for physical activity and healthy eating**										
	Perception of ability and confidence for healthy eating and exercise		4		.93		If I use an app with the abovementioned characteristics, I will be able to exercise regularly in the next 12 weeks		1=strongly disagree; 7=strongly agree	
	Perception of ability to maintain healthy eating and exercise habits		2		.87		This app would help me to maintain healthy eating		1=strongly disagree; 7=strongly agree	
**Motivation, barriers, and solutions to eating healthily and do physical activity**										
		Motivation to eat healthily		17		.83		Enjoyment from eating healthy food		1=strongly disagree; 7=strongly agree
		Barriers to eating healthily		14		.91		Lack of professional guidance		1=strongly disagree; 7=strongly agree
		Solutions to eating healthily		19		.82		Set small goals		1=strongly disagree; 7=strongly agree
		Motivation to do physical activity and exercise		15		.83		Enjoyment from physical activity or exercise		1=strongly disagree; 7=strongly agree
		Barriers to physical activity and exercise		14		.90		Lack of professional guidance		1=strongly disagree; 7=strongly agree
		Solutions to physical activity or exercise		20		.86		Set small goals		1=strongly disagree; 7=strongly agree
Intention to use the app				2		.95		I intend to use this app in the next 6 mo		1=strongly disagree; 7=strongly agree
Willingness to pay for the app				1		—^a^		How much money are you willing to spend per month for an app that combines the features mentioned above?		Answer in pound (£) and pence
Healthy lifestyle				4		.85		Following a healthy lifestyle is really important to me especially in terms of physical activity or regular exercise		1=strongly disagree; 7=strongly agree

^a^Not available.

### Measurements

#### App Feature Attitudes

To measure attitudes toward potential mobile app features, a questionnaire comprising 37 items was developed. Participants were asked to rate specific features (eg, “latest news and trends in eating and exercise,” “exercise tips,” and “healthy eating tips”; see Table S1 in Multimedia Appendix 1) according to how valuable they perceived these features to be on a scale from 1=*has no value at all* to 7=*extremely valuable*. Three items were removed from the analysis due to incomplete answers.

Items on the scale were produced cocreatively and systematically [56]. Drawing on data from the previous cocreation activities from the wider 2-week-long project, open-ended web-based responses were analyzed by the authors, and frequent suggestions were added to the item pool [57,58]. Experts (with expertise in behavioral economics, digital marketing, consumer behavior, and psychology) examined this list, adding further suggestions based on their own knowledge of the literature, and reworded items as appropriate. See Table S8 in Multimedia Appendix 1 for example quotations from the cocreative work aligned with each of the questionnaire items.

#### Reward Attitudes

To measure attitudes toward rewards for achieving goals, participants were asked what form of rewards (“gift vouchers”; “prizes like books, watches, Fitbit, and sports equipment”; “points that could be redeemed for experiences, shopping, and days out”; “discount on your shopping”; and “points can be redeemed for charitable causes”) they would like to receive from a mobile app about healthy food and exercising. These rewards differ in a number of ways: in contrast to other rewards, “points that can be redeemed for charitable causes” is a nonmonetary reward, as allowing participants to exercise for a social group would increase their motivation and engage them more with physical activity. In the experimental literature, spending money on others can lead to higher satisfaction than spending money on oneself [59]. In addition, nonmonetary or prosocial incentives could increase workers’ satisfaction and improve their performance [60]. Among the 4 monetary rewards, one is a very close substitute to cash (“discount on your shopping”), whereas the 3 others are associated with gratification and leisure (“gift vouchers”; “prizes like books, watches, Fitbit, and sports equipment”; and “points that could be redeemed for experiences, shopping and days out”).

#### Self-Efficacy for Physical Activity and Healthy Eating

To measure self-efficacy regarding physical activity and healthy eating, we used the following 2 self-designed scales: “perception of ability and confidence for healthy eating and exercise” and “perception of ability to maintain healthy eating and exercise habits.” To evaluate participants’ perceptions of ability and confidence, we asked 4 questions about using the described mobile app to track their healthy eating and exercise habits (eg, “If I use an app with the abovementioned characteristics, I will be able to exercise regularly in the next 12 weeks.*”*). To examine “perceptions of their ability to maintain healthy eating and exercise habits,*”* we asked the participants to evaluate the extent to which they agree that the app would help them to maintain healthy eating and physical activity (eg, “This app would help me to maintain healthy eating”).

#### Motivation, Barriers, and Solutions to Eating Healthily and Do Physical Activity

Items of the “motivation,” “barrier,” and “solutions” scales were produced cocreatively and systematically in the same way as the abovementioned attitude scale from an English-speaking web-based community. In 17 questions, participants were asked to indicate to what extent the factors (eg, “enjoyment from eating healthy food”) motivate them to pursue a healthy diet, and in 15 questions, they were asked to indicate to what extent the factors (eg, “enjoyment from physical activity/exercise”) motivate them to do regular physical activity and exercises.

In addition, in 14 questions, participants were asked to indicate the extent to which the barriers (eg, “lack of professional guidance” and “I lack self-control”) hinder them from pursuing a healthy diet, and in 14 questions, they were asked to indicate to what extent the barriers hinder them from doing regular physical activity and exercises.

Moreover, in 19 questions, participants were asked to indicate how the solutions (eg, “set small goals” and “pick healthy food that I like”) help them have a sustainable healthy eating, and in 20 questions, they also were asked to indicate how the solutions help them have sustainable physical activity and exercises.

#### Intention to Use and Willingness to Pay for the App

To measure participants’ intention to use an app, we asked them to answer 2 questions (eg, “I intend to use this app in the next six months”). To measure participants’ willingness to pay for this type of mobile app for healthy eating and exercise, we asked them to indicate the amount of money (in pounds sterling and pence or in euros and cents) they would be willing to spend per month for an app that combined the features mentioned earlier in the survey.

#### Healthy Lifestyle Scale

In 4 items (eg, “following a healthy lifestyle is really important to me especially in terms of physical activity/regular exercise”), participants were asked to indicate their commitment to a healthy lifestyle.

### Data Analysis

The data were analyzed using SPSS (version 25; IBM Corp) [61]. To explore the primary aim of understanding consumer preferences for mobile app features and rewards, we used the rank case method [62]. Exploratory factor analysis (EFA) was implemented using the maximum likelihood method to reduce numerous individual mobile health app features to a smaller number of key categories as perceived by consumers. We performed a parallel analysis to accept only the number of factors that exceeded the random data [63,64]. To explore the third aim of understanding whether people’s app feature attitudes would predict their intention to use and willingness to pay for it, 2 backward regression analyses were conducted to test which factors of the attitude scale predicted intention to use and willingness to pay. To examine whether BMI and self-efficacy would moderate these analyses, we investigated the interactions between attitude factors and BMI and self-efficacy to predict intention to use and willingness to pay for the app. Finally, to explore the fourth aim of investigating the characteristics of different target groups of consumers and their responses toward the app features, we used k-means cluster analysis to classify consumers into different groups and to understand which groups of consumers prefer what types of mobile app features. Cluster analysis also contributed to understanding which groups of consumers had a greater intention to use and willingness to pay for the app. To conduct the cluster analysis, we standardized all variables (*Z* scores). The k-means procedure identifies relatively homogenous subgroups while maximizing variability between clusters.

## Results

### Ranking the Preferences of Mobile App Features Based on Consumers’ Attitudes

Rank case analyses were conducted to identify the most preferred features and rewards for mobile apps designed to support healthy eating and physical activity. The results of these analyses are presented in Table S9 in Multimedia Appendix 1. The most preferred app features were “suggesting home workouts (no equipment required),” “exercise tips,” and “show your progress in graphs and charts,” and the least preferred ones were “connected to Facebook, Twitter, Insta etc”; “connected to close ones”; and “challenges with close ones.” The most preferred rewards were “gift vouchers” and “discount on your shopping,” and the least preferred was “charitable causes.”

### Factor Analysis on the Scale Measuring App Feature Attitudes

An EFA was conducted to reduce numerous individual mobile health app features to a smaller number of key categories as perceived by consumers. The results of the EFA based on parallel analysis produced 4 factors (Table 3). The numbers serve as indicators of the loading level for each item with one of the factors. Items with the highest loading for a specific factor were considered part of that factor*.* The analysis revealed that the strongest level of loading for factor 1 was related to the item “challenges with close ones” (factor loading=0.794), that for factor 2 is associated with the item “suggest quick workouts” (factor loading=0.651), that for factor 3 is related to the item “provide recipe suggestions according to your shopping list” (factor loading=0.753), and that for factor 4 is associated with the item “connected to supermarket” (factor loading=0.745). We discarded the items (“rewards for healthy eating” and “provide location of local producers”), which indicated high loading on >1 factor, as they could confound the interpretation of factors. The 4 factors included items that measured consumers’ attitudes toward app features. For instance, factor 1 (“social support, connectedness, and mindfulness”) includes items like *“*challenges with close ones” and “community support*,*” which mainly relate to social interactions. Factor 2 (“goal setting, tracking, and advice for exercising”) includes items like “suggest quick workouts” and “set regular goals*,*” which mostly relate to setting goals and planning and monitoring progress. Factor 3 (“tips and advice for food and home workouts*”*) includes items like “meal planning advice” and “healthy eating tips*,*” which mainly refer to personalized professional nutrition and exercise support, and factor 4 (“digital score connection and mood management”) includes items like “scanner for supermarket receipts” and “provide advice based on your mood*,*” which mostly relate to integrated mood-based shopping regarding food and activity.

To investigate the validity of the scale, we conducted confirmatory factor analysis (see Figure S1 and Table S10 in Multimedia Appendix 1). The results indicated a satisfactory and good model fit: *χ^2^*_393_=528.4, *P*<.001; CFI=0.958; TLI=0.947; RMSEA=0.041.

**Table 3:** Factor loadings of items of app features.

		Factors			
		1^a^	2^b^	3^c^	4^d^
**Factor loading**					
	Challenges with close ones	*0.794* ^e^	0.225	0.014	0.149
	Community support	*0.753*	0.094	0.201	0.123
	Connected to close ones	*0.749*	0.155	0.051	0.135
	Match you to app users in similar situation as you	*0.748*	0.137	0.170	0.077
	Competitions among users	*0.655*	0.248	0.046	0.201
	Connected to Facebook, Twitter, Instagram etc	*0.607*	0.039	0.113	0.251
	Latest news and trends in eating and exercise	*0.506*	0.210	0.365	0.130
	Emotional or moral support from a professional	*0.424*	0.173	0.261	0.348
	Mindfulness, yoga, and meditation (short clips)	*0.415*	0.252	0.126	0.120
	Suggest quick workouts	0.009	*0.651*	0.238	0.103
	Reminders	0.293	*0.650*	0.125	0.201
	Set regular goals (daily, weekly, or monthly)	0.106	*0.642*	0.396	0.128
	Motion sensor (to detect your activity level)	0.166	*0.639*	0.019	0.374
	Show your progress in graphs and charts	0.247	*0.626*	0.145	−0.087
	Provide a step-by-step plan for eating and exercise	0.140	*0.565*	0.500	0.130
	Exercise tips	0.209	*0.554*	0.352	0.046
	Set goals for you	0.144	*0.537*	0.250	0.174
	Motivational messages	0.496	*0.524*	0.023	0.111
	Rewards for healthy eating	0.289	*0.456*	0.096	0.456
	Connected to running apps (Strava, Fitbit)	0.283	*0.411*	0.163	0.283
	Reward for trying rather than succeeding	0.275	*0.392*	−0.195	0.373
	Provide recipe suggestions according to your shopping list	−0.008	0.152	*0.753*	0.355
	Meal planning advice	0.135	0.307	*0.728*	0.169
	Healthy eating tips	0.120	0.350	*0.699*	−0.073
	Personalized recipes	0.111	0.129	*0.693*	0.331
	Planner and tracker of your eating and exercise	0.133	0.528	*0.566*	0.082
	Sharing and exchanging recipes	0.427	0.031	*0.539*	0.121
	Guidance from a professional (dietician or fitness coach)	0.399	0.217	*0.485*	0.065
	Suggest home workouts (no equipment required)	0.125	0.417	*0.436*	0.091
	Connected to supermarket (for grocery shopping)	0.199	0.037	0.238	*0.745*
	Mood detector (suggest food and activity according to your mood)	0.192	0.258	0.137	*0.706*
	Scanner for supermarket receipts	0.147	0.032	0.146	*0.690*
	Provide advice based on your mood	0.189	0.301	0.151	*0.666*
	Provide location of local producers	0.193	0.030	0.489	*0.491*
Variance (%)		14.91	14.704	13.313	10.407
Cronbach α^f^		.868	.864	.860	.790

^a^F1: social support, connectedness, and mindfulness.

^b^F2: goal setting, tracking, and advice for exercising.

^c^F3: tips and advice for food and home workouts.

^d^F4: digital score connection and mood management.

^e^Items that are highly associated with a specific factor and exhibit a higher loading under that factor compared with other factors are italicized.

^f^Cronbach α analyses for the factors were conducted after removing the items “rewards for healthy eating*”* and *“*provide the location of local producers.”

### Backward Regression Analyses for Intention to Use an App and Willingness to Pay for an App

Two backward regression analyses were conducted to investigate whether people’s app feature attitudes predicted their intention to use and willingness to pay for it. In addition, interactions between attitude factors and BMI and self-efficacy were examined to determine whether BMI and self-efficacy would moderate these analyses, specifically predicting intention to use and willingness to pay for the app.

Backward stepwise regression analysis for intention to use resulted in the final model shown in Table 4. The results showed that “digital score connection and mood management” (β=.219; *P*<.001), “perception of ability and confidence” (β=.182; *P*=.006), and “perception of ability to maintain healthy eating and exercise habits” (β=.602; *P*<.001) were significant predictors of intention to use the app. Other explanatory variables included in the original model were dropped from the final model due to their lack of significance. The model also controlled for demographic variables such as age, gender, number of households, income, family status, country, and education.

**Table 4:** Summary of backward stepwise regression analysis for variables predicting intention to use the app^a^.

Predictor	B (SE)	β	*P* value
Constant	−2.546 (0.661)	—^b^	<.001
Digital score connection and mood management	0.120 (0.025)	.219	<.001
Health confidence^c^	0.119 (0.043)	.182	.006
Health maintenance^d^	0.724 (0.079)	.602	<.001
Social support, connectedness, and mindfulness × BMI	0.359 (0.125)	.114	.005
Goal setting, tracking, and advice for exercising × health confidence	0.656 (0.187)	.315	.001
Tips and advice for food and home workouts × health confidence	−0.671 (0.201)	−.294	.001

^a^*R*^2^=0.722, adjusted *R*^2^=0.713, *F*_6,186_=80.417, *P*=.001.

^b^Not available.

^c^Health confidence: perception of ability and confidence for healthy eating and exercise.

^d^Health maintenance: perception of ability to maintain healthy eating and exercise habits.

The results also indicated that the interaction between the “social support, connectedness, and mindfulness” factor and BMI (β=.114; *P*=.005) was a significant predictor of intention to use the app. In addition, the interaction between the “goal setting, tracking, and advice for exercising” factor and “perception of ability and confidence” (β=.315; *P*=.001) was a significant predictor of intention to use the app. Furthermore, the interaction between the “tips and advice for food and home workouts” factor and “perception of ability and confidence” (β=−.294; *P*=.001) negatively predicted intention to use the app. These results indicate that BMI and self-efficacy moderate the relationship between some factors of app feature attitudes and the intention to use the app. Specifically, the results emphasize that the factors influencing an individual’s intention to use an app that support healthy eating and physical activity depend on specific individual characteristics. The findings revealed that not only the relationship between “social support, connectedness, and mindfulness” and intention to use the app but also the link between “goal setting, tracking, and advice for exercising” and intention to use the app was stronger among people with higher BMI and high levels of “perception of ability and confidence,” respectively. These findings highlight the importance of customizing the “social support, connectedness, and mindfulness” and “goal setting, tracking, and advice for exercising” app features to meet the needs and preferences of consumers with higher BMI and high levels of self-efficacy. By contrast, the relationship between “tips and advice for food and home workouts” and intention to use the app was found to be stronger among individuals with lower levels of “perception of ability and confidence.” These findings highlight the importance of customizing the app features related to “tips and advice for food and home workouts” to meet the needs and preferences of consumers with lower levels of self-efficacy.

In summary, the results demonstrated that having a high BMI was associated with a higher impact of consumers’ attitudes toward “tips and advice for exercising” on their intention to use the app. In addition, high self-efficacy was associated with a higher impact of consumers’ attitudes toward “goal setting, tracking, and advice for exercising” and a lower impact of consumers’ attitudes toward “tips and advice for food and home workouts” on their intention to use the app. These findings highlight the important impact of consumers’ attitudes on their app use intentions. The findings also underscore the determinant roles of BMI and self-efficacy in the link between consumers’ attitudes and their intention to use the app.

The backward stepwise regression analysis for willingness to pay resulted in the final model shown in Table 5. The results showed that “social support, connectedness, and mindfulness” (β=.229; *P*=.004) and “perception of ability to maintain healthy eating and exercise habits” (β=.237; *P*=.003) were significant predictors of willingness to pay for the app. Other explanatory variables included in the original model, such as 3 of the attitude app feature factors (intention to use the app, BMI, self-efficacy, and their interaction with attitude app feature factors), were dropped from the final model due to their lack of significance. The model was also controlled for demographic variables. These results did not show that BMI and self-efficacy moderate the relationship between some factors of app feature attitudes and willingness to pay for the app.

**Table 5:** Summary of backward stepwise regression for variables predicting willingness to pay for the app^a^.

Predictor	B (SE)	β	*P* value
Constant	−2.895 (1.252)	—^b^	—
Social support, connectedness, and mindfulness	0.093 (0.032)	.229	.004
Health maintenance^c^	0.410 (0.138)	.237	.003

^a^*R*^2^=0.169, adjusted *R*^2^=0.158, *F*_2,190_=19.002, *P*<.001.

^b^Not available.

^c^Health maintenance: perception of ability to maintain healthy eating and exercise habits.

### Cluster Analysis

K-means cluster analysis was used to classify consumers into different groups and to understand which group of consumers prefer what types of mobile app features, their intention to use the app, and willingness to pay for it (Table S11 in Multimedia Appendix 1). To conduct the cluster analysis, in line with the existing literature and similar studies [65], we included all demographic and geographic variables (eg, age, gender, education, income, number of households, family status, BMI, and country), as well as psychological and behavioral factors (healthy lifestyles, experience and knowledge about health apps, self-efficacy, motivation, attitude toward health apps, and intention and willingness to pay) in the clustering. All variables were standardized (*Z* scores). The k-means procedure identifies relatively homogenous subgroups while maximizing variability between clusters. K-means cluster analysis (Table S11 in Multimedia Appendix 1) indicated a 2-cluster solution for the data.

The results (Table 6; Tables S11 and S12 in Multimedia Appendix 1) showed that consumers in cluster 2 (motivated health app enthusiasts) were younger, had smaller household numbers, and had more previous experience and knowledge about using mobile health apps. They also indicated higher motivation, higher self-efficacy, and more positive app feature attitudes and showed greater intention to use an app and pay for it than the consumers in cluster 1 (low health app users).

**Table 6:** Mean values and SDs of classification variables in clusters 1 and 2^a^.

Variables		Cluster 1 (low health app users; n=58), mean (SD)	Cluster 2 (motivated health app enthusiasts; n=133), mean (SD)
**Demographic**			
	Age^b^	37.79 (7.142)	34.01 (6.861)
**Health factors**			
	Height	173.52 (8.828)	171.82 (9.366)
	Weight	75.784 (21.834)	74.788 (15.548)
	BMI	25.056 (6.301)	25.238 (4.39)
	Health/activity^b,c^	23.137 (5.877)	26.045 (5.828)
**Motivation, barriers, and solutions**			
	Motivation-EAT^b,d^	68.051 (11.819)	80.624 (12.514)
	Barrier-EAT^e^	45.569 (16.959)	49.36 (16.002)
	Solution-EAT^b,f^	75.362 (9.995)	93.969 (12.153)
	Motivation-PHYSIC^b,g^	56.844 (11.108)	69.939 (11.621)
	Barrier-PHYSIC^h^	49.241 (16.992)	50.082 (16.481)
	Solution-PHYSIC^b,i^	78.586 (14.462)	98.864 (14.059)
**App feature attitude**			
	F1: social support, connectedness, and mindfulness^b^	23.931 (8.869)	38.894 (10.040)
	F2: goal setting, tracking, and advice for exercising^b^	47.172 (12.042)	62.36 (7.086)
	F3: tips and advice for food and home workouts^b^	34.5 (9.169)	45.533 (6.427)
	F4: digital score connection and mood management^b^	12.793 (12.787)	19.639 (5.254)
**Rewards attitude**			
	Rewards: vouchers^b^	4.90 (1.813)	5.92 (1.087)
	Rewards: prizes^b^	4.43 (1.948)	5.88 (1.135)
	Rewards: experience^b^	4.67 (1.7)	5.73 (1.262)
	Rewards: discount^b^	4.83 (1.656)	5.89 (1.024)
	Rewards: charitable^b^	4.07 (1.909)	5.23 (1.430)
**Self-efficacy**			
	Health confidence^b,j^	15.586 (5.522)	21.902 (3.457)
	Health maintenance^b,k^	7.396 (3.071)	11.218 (1.597)
**Use or pay**			
	Intention^b,l^	5.689 (2.903)	10.947 (1.982)
	Pay^b,m^	2.092 (2.417)	5.436 (5.234)

^a^The analysis was performed based on standardized (*Z*) scores.

^b^*P*<.01 (shows significant differences between the cluster centers of clusters 1 and 2 in the specific variable; refer to Table S11 in Multimedia Appendix 1 for more details on the cluster centers and *t* test results).

^c^Health/Activity: Healthy Lifestyle scale.

^d^Motivation-EAT: motivation to eat healthily.

^e^Barrier-EAT: barriers to eating healthily.

^f^Solution-EAT: solutions to eating healthily.

^g^Motivation-PHYSIC: motivation to do physical activity and exercise.

^h^Barrier-PHYSIC: barriers to physical activity and exercise.

^i^Solution-PHYSIC: solutions for physical activity or exercise.

^j^Health confidence: perception of ability and confidence for healthy eating and exercise.

^k^Health maintenance: perception of ability to maintain healthy eating and exercise habits.

^l^Intention: intention to use the app.

^m^Pay: willingness to pay for the app.

Table S11 and Figure S2 in Multimedia Appendix 1 display the contribution of each variable to cluster formation, given by effect size η2. The findings indicated that intention to use the app had the greatest contribution to cluster membership (η2=0.566). Consistently, the results of the backward regression analyses indicated that cluster membership significantly predicted intention to use (β=.307; *P*<.001; Table S13 in Multimedia Appendix 1). In addition, the factors related to app features (“goal setting, tracking, and advice for exercising*”* [η2=0.422] and “social support, connectedness, and mindfulness*”* [η2=0.372]) as well as “perception of ability to maintain healthy eating and exercise habits” (η2=0.441) and “solutions to eating healthily*”* (η2=0.393) highly contributed to the cluster membership. These results also showed that the effect size in the features related to “digital score connection and mood management*”* were lower than that in other mobile app features; this showed that differences between 2 clusters on this factor were smaller than those between other mobile app features. This suggests that consumers of both clusters might be more interested in these app features than in other features.

Moreover, the cluster analysis results did not show significant differences in cluster membership between countries (*t*_189_=1.95; *P*=.052). However, to further explore differences between countries in attitudes toward app features and intention to use, we conducted a 1-way ANOVA (Table S14 in Multimedia Appendix 1) as an exploratory analysis. The results indicated a significant effect of country on the intention to use and attitude toward app factors. Post hoc comparisons using the Bonferroni test indicated a significant difference in the intention to use the app between the United Kingdom (mean 10.098, SD 3.238) and France (mean 8.137, SD 3.638; mean difference 1.96, SE 0.66; *P*=.02). The United Kingdom (mean 37.627, SD 11.596) and France (mean 30.509, SD 12.389) were also significantly different (mean difference 7.11, SE 2.32; *P*=.01) in terms of the factor “social support, connectedness, and mindfulness.” In addition, there were significant differences (mean difference −4.78, SE 1.69; *P*=.03) between Germany (mean 40.37, SD 9.262) and Italy (mean 45.156, SD 8.261) in terms of the factor “tips and advice for food and home workouts.” There were also significant differences (mean difference −4.66, SE 1.72; *P*=.04) between France (mean 40.49, SD 9.109) and Italy (mean 45.156, SD 8.261) in the same factor. Furthermore, there were significant differences (mean difference 4.54, SE 1.13; *P*<.001) between the United Kingdom (mean 20.058, SD 5.19) and France (mean 15.509, SD 5.961) in terms of the factor “digital score connection and mood management.” Moreover, the United Kingdom (mean 20.058, SD 5.19) and Germany (mean 16.648, SD 6.237) significantly differed (mean difference 3.14, SE 1.12; *P*=.01) in the factor “digital score connection and mood management.” It is important to note that these findings on country differences are exploratory in nature, and the sample size is not sufficiently large to draw definitive conclusions regarding cross-national differences.

## Discussion

### Principal Findings

Mobile app features for an app targeting healthy eating and physical activity were cocreated by participants in a web-based community. The primary aim was to uncover consumers’ most preferred features and rewards for the future design of mobile apps that support healthy eating and physical activity. We also aimed to reduce numerous individual mobile health app features to a smaller number of key categories as perceived by consumers. Further study aims were to investigate the effect of differences in BMI and self-efficacy on the intention to use and willingness to pay for such an app. Finally, we sought to determine the characteristics of different target groups of consumers and their responses toward app features via cluster analysis. The study results indicated that app features related to “home workouts (no equipment required)*”* and “exercise tips,” as well as displaying “progress in graphs and charts*”* were the most preferred in a group of adult consumers. These results are consistent with the findings of Frontini et al [5] who revealed that healthy food and physical activity suggestions were the most important features for their sample when considering a mobile app to enhance health behaviors and physical exercise. In their study, tips and plans were the most popular features of a mobile health app. This study explored these elements further by specifying the type and location for performing physical activity; participants demonstrated a preference for workouts at home. Interestingly, participants preferred to undertake “home workouts with no need for equipment,” which can make physical activity and exercise more feasible [66]. In addition, quick workouts were preferred. Our results also highlight consumers’ preference for tracking their own progress using graphs and charts. Individuals also indicated that *“*connections to close ones*”* and “connection to Facebook, Twitter, and Instagram” were less important; consumers had appeared to prefer features that provided feasible, activity-based feedback in intervention programs over those that provide interaction with close ones or broader society on social media platforms. This is also consistent with the findings of Frontini et al [5], who suggested a lack of privacy and personal exposure as one of the reasons why people do not use mobile health apps.

The study findings also showed that “gift vouchers,” which referred to vouchers from food stores, was the most preferred reward, followed by “discounts on shopping*”* and *“*prizes like books, watches, Fitbit, and sports equipment.” This highlights the potential role of monetary rewards in promoting exercise and healthy lifestyles as opposed to charity rewards. Moreover, the fact that “gift vouchers” were preferred over “discounts on shopping” shows that participants valued the possibility of securing an indulgent reward versus the freedom of a closer to a cash type of reward. This study also took an intermediate step in simplifying the various features of mobile health apps by conducting an EFA. The results showed that the main components and key categories of mobile apps combining healthy eating and exercise are “social support, connectedness, and mindfulness”; “goal setting, tracking, and advice for exercising”; “tips and advice for food and home workouts”; and “digital score connection and mood management.” These findings may help organize app features into key components and categories. This corresponds with some recent efforts [67] that have shown the dimensions of app features in clinical domains to help health experts in the diagnosis process. The dimensions mentioned in the recent studies [67] are fulfilling consumers’ short-term and long-term needs (usefulness: functionality and information quality); apps’ ease of use (usability: guidance, social sharing, and tutorial); and “trust app features (privacy, security, and reliability).” Importantly, the results of this study contribute to the growing body of knowledge supporting the construction of effective mobile apps that aim to enhance health behaviors not only in clinical population but also in general public [68].

We also found that app feature attitudes were associated with intention to use and willingness to pay for an app. Interestingly, specific positive attitudes around “social support, connectedness, and mindfulness” were strongly associated with willingness to pay for the app. The results suggest that although some items of “social support, connectedness, and mindfulness” (eg, “connections to close ones” and “connection to Facebook, Twitter, and Instagram”) were among the least important app features, people were still willing to pay for an app that included those features*.* Furthermore, the results showed that the relationship between consumers’ attitude toward “social support, connectedness, and mindfulness” as a feature and their intention to use the app was stronger for individuals with higher BMI than for those with lower BMI. High self-efficacy was also associated with more positive attitudes toward “goal setting, tracking, and advice for exercising*”* feature of the app, in addition to intention to use the app. These findings are consistent with those of previous studies on social cognitive theory and health enhancement, which indicate an association between motivation, self-efficacy, and behavioral intentions [69,70]. Previous studies have also shown that self-efficacy is related to higher levels of exercise [71] and is influential in supporting people to overcome barriers to physical activity [72].

The results indicated that higher self-efficacy was related to a lower positive relationship between attitude toward “Tips and advice for food and home workouts*”* app feature and intention to use the app; that is, those with high self-efficacy might be less likely to use the app with this feature. Bandura [69] stated that self-efficacy could also negatively impact motivation and intention when high self-efficacy causes individuals to think that they are sufficiently capable of achieving a goal. Likewise, Vancouver et al [73] and Zhang [46] claimed that high self-efficacy might reduce the expected resource needs to reach goals, as self-efficacy reduces the subjective evaluation of the discrepancy between the goal and reality. Therefore, the negative impact of self-efficacy on the relationship between attitude and intention to use the app in this study may indicate that consumers with high levels of self-efficacy are confident that they are sufficiently capable of achieving their health goals without the need for external help. Future research should continue to investigate whether this group might need different types of support and how self-efficacy impacts consumers’ motivation and intention when goal perceptions vary.

With the objective of exploring group characteristics, the novelty of this study was the cluster analysis, which considered the diverse demographic, geographic, psychological, and behavioral factors of consumers in the United Kingdom, Germany, France, and Italy. The results showed 2 clusters and indicated that people in cluster 2 (motivated health app enthusiasts) who were younger, had smaller household numbers, and had more experience and knowledge about using mobile health apps were more motivated to use the app and had a more positive app feature attitude, indicating more intention to use and willingness to pay for the app than the consumers in cluster 1 (low health app users). The results showed that 75.9% (145/191) of participants (Table S12 in Multimedia Appendix 1) reported having prior information and experience of using mobile apps for healthy eating and physical activity. In addition, people in cluster 2 (motivated health app enthusiasts) had more prior experience and knowledge about using mobile health apps than cluster 1 (low health app users). The results showed smaller differences between the 2 clusters in the features related to “digital score connection and mood management, ” even though these differences were significant. This suggests that consumers from both clusters might share an interest in these features compared with others. In addition to helping consumers gain more experience and knowledge in using mobile health apps to enhance their target users’ experience, marketers and app designers should prioritize features that assist their consumers. For instance, they should include elements that enhance consumers’ social support, connectedness, and mindfulness. Furthermore, users should be empowered to set and track their dietary and physical activity goals more effectively, receive simplified advice for meals and workouts, and manage their mood better through improved gamification systems. As a result, these findings hold practical implications for future app development by highlighting subgroup needs and attitudes; the results can aid marketers, app designers, and experts in health-related research to identify target groups of consumers interested in specific features of mobile health apps.

Although the exploratory analysis indicated some differences in intentions to use the app and attitudes toward app factors among countries, the cluster analysis did not show significant differences in cluster membership. This emphasizes the need for further research with larger sample sizes to determine countries’ differences in cluster membership. Furthermore, exploring how different clusters within these countries respond differently to the intention to use the app and the factors related to attitude toward app features can have practical implications for future app development, facilitating the customization of apps to meet the specific needs of consumers in these countries.

### Limitations and Future Research

A limitation of this study is that although our sample is diverse, it was recruited through a web-based platform and thus might have included fewer participants with lower education levels and less prior knowledge and experience in using mobile health apps. For instance, three-quarters of the participants mentioned having previous information and experience of using mobile apps for healthy eating and physical activity. Most participants were also highly educated (bachelor’s degree or higher: 145/191, 75.9%) and might therefore be more comfortable using mobile apps than the general population [74]. Future studies should include more participants with lower education levels and those with less prior knowledge and experience of using mobile health apps. Similar to the sample used by Lee and Cho [75] in their study, our sample was sufficiently robust to conduct a survey on individuals’ attitudes toward app features. However, to achieve higher external validity and facilitate cross-national comparisons, future studies should aim to increase the number of participants.

### Conclusions

This study has demonstrated that feasible, activity-based features (eg, *“*suggesting home workouts” and “exercise tips”) and monetary rewards (eg, “gift vouchers”) were the most preferred mobile health app features and rewards, respectively, in a sample from 4 European countries. The study reduced the number of mobile health app features as suggested by the participants and experts to 7 main components and categories. The findings also highlight the impact of differences in the health status of consumers and relevant motivational factors on app feature preferences.

Finally, the results suggest that consumers’ motivational factors, basic demographics (age and household number), and socioeconomic status lead to different attitudes toward app features and cause individuals to show different levels of intention to use and willingness to pay for those features. This study contributes to a better understanding of consumers who might form an appropriate target market for marketers and app designers producing mobile apps that are aimed at improving healthy eating and exercise in the general population.

## Appendix

Multimedia Appendix 1 Supplementary materials such as tables and figures, which further explain the measurements and findings of the study.

## Abbreviations

EFA: exploratory factor analysis
