# Supplementary material for: Unveiling Consumer Preferences and Intentions for Cocreated Features of a Combined Diet and Physical Activity App: Cross-Sectional Study in 4 European Countries
Source: JMIR Hum Factors. 2023 Dec 11;10:e44993. doi: 10.2196/44993 (PMC10750232; doi:10.2196/44993)
Supplement: Multimedia Appendix 1 [file humanfactors_v10i1e44993_app1.docx]

**Supplementary materials**

**Measurements**

***App feature attitude***

The attitude towards mobile app features scale (See Table S1) included 37 items. We told participants that we are in the process of designing an app to motivate people to eat healthily and exercise regularly. Hereafter, we asked them to rate the features of the app (See Table S1) according to how valuable they find them on a scale from 1= *has no value at all* to 7 = *extremely valuable*. Items 27, 27A and 27B were removed from the analysis because of _incomplete answers.

***Reward Attitude***

We asked participants, what form of rewards they would like to receive from a mobile app about healthy food and exercising which provided rewards for achieving goals. In five questions (See Table S1), we asked them to indicate their answers on a scale from 1= *strongly disagree* to 7 = *strongly agree*.

Table S1

*Items of the scales* *App feature attitudes and Rewards attitudes*

| App feature attitudes | Rewards attitudes |
| --- | --- |
| 1. Latest news and trends in eating and exercise | 1. Gift vouchers |
| 1. Exercise tips | 1. Prizes like books, watches, fitbit, sports equipment |
| 1. Healthy eating tips | 1. Points that can be redeemed for experiences, shopping, days out |
| 1. Meal planning advice | 1. Discount on your shopping |
| 1. Set regular goals (daily/weekly/monthly) | 1. Points can be redeemed for charitable causes |
| 1. Personalised recipes |  |
| 1. Provide a step by step plan for eating and exercise |  |
| 1. Sharing and exchanging recipes |  |
| 1. Connected to Facebook, twitter, insta etc |  |
| 1. Connected to close ones |  |
| 1. Community support |  |
| 1. Connected to supermarket (for grocery shopping) |  |
| 1. Provide recipe suggestions according to your shopping list |  |
| 1. Provide location of local producers |  |
| 1. Rewards for healthy eating |  |
| 1. Competitions among users |  |
| 1. Reminders |  |
| 1. Planner and tracker of your eating and exercise |  |
| 1. Show your progress in graphs and charts |  |
| 1. Connected to running apps (strava, fitbit) |  |
| 1. Provide advice based on your mood |  |
| 1. Set goals for you |  |
| 1. Match you to app users in similar situation as you |  |
| 1. Motion sensor (to detect your activity level |  |
| 1. Scanner for supermarket receipts |  |
| 1. Mood detector (suggest food and activity according to your mood) |  |
| 1. Set targets and offer feedback |  |
| 27A. Goals set by a team of mentors |  |
| 27B. Get feedback from a team of mentors |  |
| 1. Suggest quick workouts |  |
| 1. Suggest home workouts (no equipment required) |  |
| 1. Mindfulness, yoga and meditation (short clips) |  |
| 1. Reward for trying rather than succeeding |  |
| 1. Guidance from a professional (dietician/ fitness coach) |  |
| 1. Emotional/moral support from a professional |  |
| 1. Motivational messages |  |
| 1. Challenges with close ones |  |

***Self-efficacy for physical activity and healthy eating***

We used two different measurements (see Table S2) to evaluate people’s self-efficacy regarding physical activity and healthy eating.

*‘Perception of ability and confidence for healthy eating and exercise’*. We asked participants what they thought about using the described mobile app to track their healthy eating and exercising habits. We asked participants to answer four questions on a scale from 1= *strongly disagree* to 7 = *strongly agree*.

*‘Perception of ability to maintain healthy eating and exercise habits’*. This is another self-efficacy measurement that we used to evaluate the extent which participants agree that the app would help them to maintain healthy eating and physical activity. We asked participants to indicate their agreement with the statements on a scale from 1= *strongly disagree* to 7 = *strongly agree*.

Table S2

*Items of the self-efficacy scales*

| Perception of ability and confidence for healthy eating and exercise | Perception of ability to maintain healthy eating and exercise habits |
| --- | --- |
| 1. If I use an app with the above-mentioned characteristics, I will be able to exercise regularly in the next 12 weeks. | 1. This app would help me to maintain healthy eating |
| 1. If I use an app with the above-mentioned characteristics, I will be confident over the next 12 weeks I could overcome obstacles that prevent me from exercising regularly. | 1. This app would help me maintain physical activity and exercise |
| 1. If I use an app with the above-mentioned characteristics, I will be able to eat healthily in the next 12 weeks. |  |
| 1. If I use an app with the above mentioned characteristics, I will be confident over the next 12 weeks I could overcome obstacles that prevent me from eating healthily. |  |

***Motivation to eat healthily and do physical activity*** *(These scales are reported in Snuggs et al. 2022)*

In seventeen questions (see Table S3), we asked participants to indicate to what extent the factors motivate them to pursue a healthy diet. Similarly, in fifteen questions (see Table S3), we asked participants to indicate to what extent the factors motivate them to do regular physical activity and exercises on a scale from 1= *strongly disagree* to 7 = *strongly agree*.

Table S3

*Items of the scales motivation to eat healthily and do physical activity*

| Motivation to eat healthily | Motivation to do physical activity |
| --- | --- |
| 1. Encouragement from close ones | 1. Encouragement from close ones |
| 1. Support from close ones | 1. Support from close ones |
| 1. Pressure/ comments from close ones | 1. Pressure/ comments from close ones |
| 1. Enjoyment from eating healthy food | 1. Enjoyment from physical activity/exercise |
| 1. Looking fit | 1. Looking fit |
| 1. Weight loss | 1. Weight loss |
| 1. Impress others | 1. Impress others |
| 1. Feel healthy | 1. Feel healthy |
| 1. Constant reminders keep me motivated to eat healthily | 1. Constant reminders keep me motivated to eat healthily |
| 1. Seeing and tracking progress | 1. Seeing and tracking progress |
| 1. Setting health goals for myself | 1. Setting health goals for myself |
| 1. Guilt after eating unhealthy food | 1. Guilt after eating unhealthy food |
| 1. Shame from current state of health | 1. Shame from current state of health |
| 1. Medical advice or llness | 1. Medical advice or illness |
| 1. Take care of myself | 1. So that I can celebrate the results of exercising |
| 1. Take care of my close ones |  |
| 1. So that I can celebrate my success/result from healthy eating |  |

***Barriers to eating healthily and do physical activity*** *(These scales are reported in Snuggs et al. 2022)*

In fourteen questions (see Table S4), we asked participants to indicate the extent to which the barriers hinder them from pursuing a healthy diet. Similarly, using the same fourteen questions (see Table S4), we asked participants to indicate to what extent the barriers hinder them from doing regular physical activity and exercises on a scale from 1= *strongly disagree* to 7 = *strongly agree*.

Table S4

*Items of the scales barriers to eating healthily and do physical activity*

| Items of the barrier scales |
| --- |
| 1. I don't have the time |
| 1. I don't have the money |
| 1. I think it takes too much effort o |
| 1. I don't have the skills |
| 1. I lack self control |
| 1. I give in to temptations |
| 1. I don't have the support of people close to me |
| 1. I'm too stressed |
| 1. I am in a bad mood |
| 1. I lack a routine |
| 1. It is difficult to change my habits |
| 1. I don't know how to go about it |
| 1. Lack of professional guidance |
| 1. I don't care about eating healthily |
|  |

***Solutions to eating healthily and do physical activity*** *(These scales are reported in Snuggs et al. 2022)*

In nineteen questions (see Table S5), we asked participants to indicate how the solutions help them have a sustainable healthy eating on a scale from 1= *strongly disagree* to 7 = *strongly agree*. Moreover, in twenty questions, we asked them to indicate how the solutions help them have sustainable physical activity and exercises on a scale from 1= *strongly disagree* to 7 = *strongly agree*.

Table S5

*Items of the scales solutions to eating healthily and do physical activity*

| Solutions to eating healthily | Solutions to do physical activity |
| --- | --- |
| 1. Set small goals | 1. Set small goals |
| 1. Set realistic expectations | 1. Set realistic expectations |
| 1. Make flexible plans | 1. Make flexible plans |
| 1. Set regular goals (daily/weekly/monthly) | 1. Set regular goals (daily/weekly/monthly) |
| 1. Seek professional advice (dietician) | 1. Seek professional advice (fitness coach) |
| 1. Install/Use a health app | 1. Use/Install a fitness app |
| 1. Keep a diary | 1. Keep a diary |
| 1. Share my recipes in a community (online/with close ones) | 1. Share my exercise routine in a community (online/with close ones) |
| 1. Make meal plans | 1. Make exercise plans |
| 1. Track my progress | 1. Track my progress |
| 1. Practice self control/willpower | 1. Practice self control/willpower |
| 1. Avoid temptation | 1. Avoid temptation |
| 1. Practice mindfulness (yoga/meditation) | 1. Mindfulness (yoga/meditation) |
| 1. Seek support from similar people | 1. Seek support from similar people |
| 1. Make healthy eating enjoyable for myself | 1. Make exercise enjoyable for yourself |
| 1. Pick healthy food that I like | 1. Pick exercises that I like |
| 1. Choose foods that provide quick results | 1. Pick exercises that are most efficient |
| 1. Set constant reminders | 1. Choose activities that provide quick results |
| 1. Reward myself or close ones | 1. Set constant reminders |
|  | 1. Reward myself or others |

***Intention to use the app***

We asked participants to indicate the extent to which they would intend to use the mobile app for healthy eating and exercise in the next six months (see Table S6). In particular, we asked them to answer two questions on a scale from 1= *strongly disagree* to 7 = *strongly agree*.

Table S6

*Items of the scales Intention to use the app*

| Intention to use the app |
| --- |
| 1. I intend to use this app in the next six months |
| 1. In the next six months I intend to use this app frequently |

***Intention to pay for the app***

We measured participants’ willingness to pay for the mobile app for healthy eating and exercise by asking them to indicate the amount of money (in Pounds sterling and pence and in euros and cent) they would be willing to spend per month for an app that combined the features mentioned earlier in the survey.

***Healthy Lifestyle scale***

We asked participants to indicate the extent to which they agree that following a healthy lifestyle is important to them (see Table S7). We asked them to indicate their commitment to a healthy lifestyle on a scale from 1= *strongly disagree* to 7 = *strongly agree*.

Table S7

*Items of the scales healthy Lifestyle scale*

| Healthy Lifestyle scale |
| --- |
| 1. Following a healthy lifestyle is really important to me (especially in terms of physical activity/regular exercise). |
| 1. Following a healthy lifestyle is really important to me (especially in terms of healthy eating ). |
| 1. Being physically active is an integral part of my daily life. |
| 1. Eating healthy food is an integral part of my daily life. |
| 1. I believe I am in good health |

**Example quotations from the co-creative work aligning with each of the questionnaire items.**

Table S8

*Example suggestions from co-creation study to support app features named in the questionnaire*

| Questionnaire item | Example quotation |
| --- | --- |
| Suggest home workouts no equipment required | *Videos, preferably without the use of gym equipment as lots of people are unable to purchase gym equipment* |
| Exercise tips | *An app service that gives exercising tips* |
| Show your progress in graphs and charts | *Have a chart of activities, or diet* |
| Suggest quick workouts | *Include 15 minutes of physical activity* |
| Healthy eating tips | *There would recipes and tips along the way* |
| Set regular goals |  |
| Set goals for you | *Maybe you could set yourself goals with your diet that you would add into an app* |
| Planner and tracker of your eating and exercise | *Keeping track of food and exercise can become addictive and makes you want to do better!* |
| Provide a step by step plan for eating and exercise | *Trained people who can make a plan for you, check up on you and encourage you* |
| Set targets and offer feedback | *By using the app to monitor extra food consumed, exercise patterns and body stats, it can suggest exercise plans* |
| Motion sensor to detect your activity level | *It can make tailored recommendations according to what goals you inputted* |
| Meal planning advice | *Monitor what you are eating throughout the day…it would create menus, recipes, shopping lists and meal planners* |
| Provide recipe suggestions according to your shopping list | *Offer recipe guidance, and a selection of possible recipes based on your shopping and the food stuffs that you can input as having in the store cupboard* |
| Personalised recipes | *Have recipes that feature these foods so you know what to buy and how to prepare them* |
| Rewards for healthy eating | *Give up cake for a month and receive vouchers for free fruit* |
| Connected to running apps strava, fitbit | *Link the app to Fitbit or similar so that it can measure and make tailored recommendations* |
| Provide advice based on your mood | *There would be a section that allowed you to monitor how you are feeling day to day* |
| Reminders | *Whilst shopping, if you are getting reminders, it's reinforcing the idea* |
| Guidance from a professional dietician/ fitness coach | *Nutritionists, personal trainers, who would be there just to help with advice and new recipes* |
| Goals set by a team of mentors | *They are able to set goals, or have them set by a trainer, which would be constantly changing and adapting to how they are progressing* |
| Mood detector suggest food and activity according to your mood | *It would be useful for someone who is doing this on their own and needs the support to continue* |
| Provide location of local producers | *In conjunction with supermarkets would make it more affordable, using wonky veg etc.* |
| Get feedback from a team of mentors | *A plethora of health and fitness professionals all inputting into your pursuit, supporting your journey* |
| Reward for trying rather than succeeding | *The person can try what they like after trying an activity, then continue to take part or start the circuit again* |
| Motivational messages | *The app could send you notifications with motivational pictures and quotes* |
| Sharing and exchanging recipes | *If you wanted to cook a healthy meal but lacked inspiration…take a photo of your fridge…other people could help, suggesting meals you could cook* |
| Emotional/moral support from a professional | *It's a network to offer moral, emotional and physical support* |
| Mindfulness, yoga and meditation short clips | *Mindfulness, breathing exercises and stretching exercises could be included* |
| Latest news and trends in eating and exercise | *Perhaps a newsletter linked to the app…it could feature interviews with people who have used it to get healthy* |
| Connected to supermarket for grocery shopping | *It could easily be incorporated into something like the Tesco Clubcard, or Sainsbury's Nectar card…it would be one of their offerings to promote healthy eating* |
| Scanner for supermarket receipts | *I keep a log of all the food I buy along with Best Before Date. How about an app which is a log of the same details, notifies you when the date is coming up?* |
| Match you to app users in similar situation as you | *The app could have some kind of community behing it which would allow you to connect with people in similar situations* |
| Community support | *People most succeed when they have support from others* |
| Competitions among users | *Generate competitions, with rewards - fun amongst family and friends* |
| Challenges with close ones | *Weekly workshops with games and challenges, and prizes* |
| Connected to close ones | *The app would geolocate you and link you with others nearby who are also looking to go out exercising* |
| Connected to Facebook, Twitter, Instagram etc | *There could be a private Facebook group, for example, where you share personal stories and struggles* |

**Ranking the preferences of mobile app features based on consumers’ attitudes**

Table S9

*Rank case analysis on mean score of attitudes towards mobile app features and the rewards for app features.*

|  | App features |  |  |  | Rewards |  |
| --- | --- | --- | --- | --- | --- | --- |
| Ranking | Items | *M* |  | Ranking |  | *M* |
| 1 | Suggest home workouts (no equipment required) | 5.98 |  | 1 | Gift vouchers | 5.56 |
| 2 | Exercise tips | 5.73 |  | 2 | Discount on your shopping | 5.55 |
| 2 | Show your progress in graphs and charts | 5.73 |  | 3 | Prizes like books, watches, fitbit, sports equipment | 5.45 |
| 4 | Suggest quick workouts | 5.65 |  | 4 | Points that can be redeemed for experiences, shopping, days out | 5.40 |
| 5 | Healthy eating tips | 5.63 |  | 5 | Points can be redeemed for charitable causes | 4.88 |
| 6 | Set regulatory goals | 5.62 |  |  |  |  |
| 7 | Set goals for you | 5.53 |  |  |  |  |
| 8 | Planner and tracker of your eating and exercise | 5.50 |  |  |  |  |
| 9 | Provide a step by step plan for eating and exercise | 5.46 |  |  |  |  |
| 10 | Motion sensor (to detect your activity level) | 5.38 |  |  |  |  |
| 11 | Meal planning advice | 5.36 |  |  |  |  |
| 12 | Provide recipe suggestions according to your shopping list | 5.36 |  |  |  |  |
| 13 | Personalised recipes | 5.19 |  |  |  |  |
| 14 | Rewards for healthy eating | 4.92 |  |  |  |  |
| 15 | Connected to running apps (strava, fitbit) | 4.86 |  |  |  |  |
| 16 | Provide advice based on your mood | 4.85 |  |  |  |  |
| 17 | Reminders | 4.83 |  |  |  |  |
| 18 | Guidance from a professional (dietician/ fitness coach) | 4.77 |  |  |  |  |
| 19 | Mood detector (suggest food and activity according to your mood) | 4.67 |  |  |  |  |
| 20 | Provide location of local producers | 4.64 |  |  |  |  |
| 21 | Reward for trying rather than succeeding | 4.60 |  |  |  |  |
| 22 | Motivational messages | 4.50 |  |  |  |  |
| 23 | Sharing and exchanging recipes | 4.49 |  |  |  |  |
| 24 | Emotional/moral support from a professional | 4.36 |  |  |  |  |
| 25 | Mindfulness, yoga and meditation (short clips) | 4.30 |  |  |  |  |
| 26 | Latest news and trends in eating and exercise | 4.29 |  |  |  |  |
| 27 | Connected to supermarket (for grocery shopping) | 4.13 |  |  |  |  |
| 28 | Scanner for supermarket receipts | 4.07 |  |  |  |  |
| 29 | Match you to app users in similar situation as you | 3.96 |  |  |  |  |
| 30 | Community support | 3.88 |  |  |  |  |
| 31 | Competitions among users | 3.62 |  |  |  |  |
| 32 | Challenges with close ones | 3.59 |  |  |  |  |
| 33 | Connected to close ones | 3.55 |  |  |  |  |
| 34 | Connected to Facebook, twitter, Instagram etc | 2.72 |  |  |  |  |

*Figure S1.* Confirmatory Factor Analysis (CFA). All pathways are shown within the model are significant (*p*<.001). F1= Social support, connectedness and mindfulness; F2= Goal setting, tracking, and advice for exercising; F3= Tips and advice for food and home workouts; F4= Digital score connection and mood management.

Table S10

*Unstandardized estimates and Standard Errors for all Covariate Paths*

| Covariance | | | Unstandardized  estimates (B) | S.E. | C.R. | sig |
| --- | --- | --- | --- | --- | --- | --- |
| F1 | <--> | F3 | 1.081 | .186 | 5.820 | <.001 |
| F1 | <--> | F2 | .652 | .120 | 5.455 | <.001 |
| F2 | <--> | F3 | .679 | .123 | 5.526 | <.001 |
| F4 | <--> | F1 | 1.307 | .231 | 5.651 | <.001 |
| F4 | <--> | F2 | .645 | .118 | 5.467 | <.001 |
| F4 | <--> | F3 | .970 | .199 | 4.871 | <.001 |
| e1 | <--> | e3 | .862 | .155 | 5.576 | <.001 |
| e30 | <--> | e32 | .857 | .197 | 4.358 | <.001 |
| e29 | <--> | e31 | .794 | .226 | 3.519 | <.001 |
| e25 | <--> | e26 | -.266 | .096 | -2.757 | .006 |
| e24 | <--> | e26 | .419 | .125 | 3.341 | <.001 |
| e22 | <--> | e23 | .235 | .071 | 3.300 | <.001 |
| e21 | <--> | e24 | .390 | .104 | 3.739 | <.001 |
| e21 | <--> | e25 | -.268 | .074 | -3.612 | <.001 |
| e20 | <--> | e23 | -.312 | .108 | -2.883 | .004 |
| e18 | <--> | e20 | .505 | .170 | 2.967 | .003 |
| e18 | <--> | e21 | -.400 | .114 | -3.501 | <.001 |
| e21 | <--> | e28 | .371 | .093 | 3.999 | <.001 |
| e19 | <--> | e31 | .486 | .172 | 2.827 | .005 |
| e18 | <--> | e31 | -.531 | .168 | -3.161 | .002 |
| e16 | <--> | e32 | .179 | .078 | 2.297 | .022 |
| e16 | <--> | e28 | .263 | .067 | 3.911 | <.001 |
| e16 | <--> | e27 | .311 | .087 | 3.557 | <.001 |
| e16 | <--> | e29 | -.266 | .098 | -2.721 | .007 |
| e16 | <--> | e23 | .176 | .059 | 2.999 | .003 |
| e15 | <--> | e24 | .232 | .083 | 2.797 | .005 |
| e14 | <--> | e28 | .284 | .079 | 3.587 | <.001 |
| e14 | <--> | e29 | -.292 | .115 | -2.533 | .011 |
| e21 | <--> | e31 | .592 | .142 | 4.164 | <.001 |
| e19 | <--> | e28 | .234 | .103 | 2.260 | .024 |
| e13 | <--> | e30 | .252 | .104 | 2.411 | .016 |
| e13 | <--> | e26 | -.409 | .130 | -3.149 | .002 |
| e13 | <--> | e22 | -.342 | .083 | -4.126 | <.001 |
| e13 | <--> | e21 | -.372 | .097 | -3.857 | <.001 |
| e13 | <--> | e15 | -.308 | .090 | -3.439 | <.001 |
| e13 | <--> | e19 | .273 | .140 | 1.943 | .052 |
| e12 | <--> | e15 | .189 | .075 | 2.527 | .012 |
| e21 | <--> | e29 | .557 | .137 | 4.073 | <.001 |
| e12 | <--> | e25 | .216 | .065 | 3.344 | <.001 |
| e12 | <--> | e17 | .152 | .071 | 2.147 | .032 |
| e11 | <--> | e18 | .290 | .130 | 2.230 | .026 |
| e10 | <--> | e29 | -.234 | .096 | -2.439 | .015 |
| e10 | <--> | e28 | .373 | .078 | 4.803 | <.001 |
| e9 | <--> | e18 | .472 | .179 | 2.635 | .008 |
| e8 | <--> | e27 | 1.150 | .171 | 6.734 | <.001 |
| e7 | <--> | e23 | .337 | .096 | 3.493 | <.001 |
| e6 | <--> | e13 | -.434 | .139 | -3.120 | .002 |
| e5 | <--> | e32 | -.214 | .107 | -2.005 | .045 |
| e5 | <--> | e26 | .392 | .138 | 2.837 | .005 |
| e5 | <--> | e10 | -.182 | .086 | -2.108 | .035 |
| e4 | <--> | e27 | .533 | .126 | 4.246 | <.001 |
| e4 | <--> | e14 | .362 | .113 | 3.191 | .001 |
| e3 | <--> | e25 | -.226 | .082 | -2.740 | .006 |
| e3 | <--> | e23 | .172 | .076 | 2.262 | .024 |
| e1 | <--> | e5 | .775 | .149 | 5.213 | <.001 |
| e24 | <--> | e29 | .373 | .120 | 3.106 | .002 |
| e14 | <--> | e27 | .261 | .107 | 2.431 | .015 |
| e12 | <--> | e24 | .218 | .074 | 2.955 | .003 |
| e10 | <--> | e21 | .242 | .082 | 2.959 | .003 |
| e10 | <--> | e19 | .276 | .103 | 2.666 | .008 |
| e10 | <--> | e16 | .165 | .067 | 2.475 | .013 |
| e6 | <--> | e31 | .551 | .190 | 2.902 | .004 |
| e2 | <--> | e5 | .354 | .149 | 2.380 | .017 |
| e2 | <--> | e13 | -.237 | .109 | -2.176 | .030 |
| e20 | <--> | e22 | -.264 | .113 | -2.341 | .019 |
| e20 | <--> | e21 | -.332 | .123 | -2.694 | .007 |
| e6 | <--> | e29 | .432 | .172 | 2.511 | .012 |
| e6 | <--> | e26 | .394 | .159 | 2.480 | .013 |
| e1 | <--> | e2 | .237 | .117 | 2.021 | .043 |
| e4 | <--> | e7 | -.291 | .138 | -2.116 | .034 |
| e2 | <--> | e4 | .596 | .161 | 3.693 | <.001 |

*Notes.* Only significant covariates are shown within the table.

Table S11

*Descriptive statistics of classification variables in Clusters 1 (Low Health App Users) and 2 (Motivated Health App Enthusiasts), and t test results for investigating differences between the two clusters.*

|  |  | Cluster 1 (N= 58) | |  | Cluster 2 (N= 133) | |  |  |  |  |
| --- | --- | --- | --- | --- | --- | --- | --- | --- | --- | --- |
| Variables |  | Cluster centers | *SD* |  | Cluster centers | *SD* |  | *t* | Sig | Effect size (*η^2^*) |
| *Demo/Geographic* |  |  |  |  |  |  |  |  |  |  |
| Age |  | .390 | 1.009 |  | -.144 | .969 |  | 3.463 | .001 | .069 |
| Gender |  | .216 | 1.033 |  | -.037 | .981 |  | 1.619 | .107 | .016 |
| Education |  | .200 | 1.091 |  | -.092 | .938 |  | 1.885 | .061 | .021 |
| Family status |  | .15598 | 1.038 |  | -.026 | .968 |  | 1.171 | .243 | .008 |
| Number of households |  | .263 | 1.122 |  | -.102 | .909 |  | 2.378 | .018 | .033 |
| Income |  | .208 | 1.106 |  | -.095 | .952 |  | 1.929 | .055 | .022 |
| Country |  | .145 | .866 |  | -.158 | 1.038 |  | 1.956 | .052 | .023 |
| *Health factors* |  |  |  |  |  |  |  |  |  |  |
| Height |  | .168 | .970 |  | -.017 | 1.029 |  | 1.172 | .243 | .008 |
| Weight |  | -.006 | .529 |  | -.030 | .377 |  | .358 | .721 | .0008 |
| BMI |  | .033 | 1.246 |  | .069 | .868 |  | -.229 | .819 | .0003 |
| Health/Activity |  | -.338 | .988 |  | .150 | .979 |  | -3.162 | .002 | .058 |
| *Previous Experience/Knowledge* |  |  |  |  |  |  |  |  |  |  |
| Using app for healthy eating |  | -.607 | .860 |  | .240 | .928 |  | -5.931 | .000 | .178 |
| Using app for physical activity |  | -.614 | .974 |  | .228 | .909 |  | -5.767 | .000 | .170 |
| *Motivation, barriers and solutions* |  |  |  |  |  |  |  |  |  |  |
| Motivation-EAT |  | -.617 | .832 |  | .268 | .881 |  | -6.491 | .000 | 0.206 |
| Barrier-EAT |  | -.108 | 1.014 |  | .117 | .956 |  | -1.479 | .141 | 0.013 |
| Solution -EAT |  | -.899 | .674 |  | .355 | .819 |  | -10.243 | .000 | 0.393 |
| Motivation-PHYSIC |  | -.684 | .842 |  | .309 | .881 |  | -7.256 | .000 | 0.245 |
| Barrier-PHYSIC |  | .002 | 1.001 |  | .051 | .971 |  | -.321 | .748 | 0.0006 |
| Solution -PHYSIC |  | -.848 | .861 |  | .360 | .837 |  | -9.087 | .000 | 0.328 |
| *App Feature Attitude* |  |  |  |  |  |  |  |  |  |  |
| F1: Social support, connectedness, and mindfulness |  | -.864 | .742 |  | .387 | .840 |  | -9.802 | .000 | .372 |
| F2: Goal setting, tracking, and advice for exercising |  | -.953 | 1.073 |  | .400 | .631 |  | -10.873 | .000 | .422 |
| F3: Tips and advice for food and home workouts |  | -.873 | 1.034 |  | .371 | .725 |  | -9.524 | .000 | .357 |
| F4: Digital score connection and mood management |  | -.821 | .834 |  | .323 | .879 |  | -8.405 | .000 | .304 |
| *Rewards Attitude* |  |  |  |  |  |  |  |  |  |  |
| Rewards: Vouchers |  | -.452 | 1.244 |  | .248 | .746 |  | -4.812 | .000 | .125 |
| Rewards: Prizes |  | -.655 | 1.248 |  | .272 | .727 |  | -6.440 | .000 | .204 |
| Rewards: Experience |  | -.485 | 1.140 |  | .223 | .846 |  | -4.768 | .000 | .123 |
| Rewards: Discount |  | -.529 | 1.208 |  | .249 | .747 |  | -5.430 | .000 | .154 |
| Rewards: Charitable |  | -.491 | 1.150 |  | .210 | .861 |  | -4.654 | .000 | .118 |
| *Self-efficacy* |  |  |  |  |  |  |  |  |  |  |
| Health Confidence |  | -.819 | 1.066 |  | .400 | .667 |  | -9.583 | .000 | .362 |
| Health Maintenance |  | -.941 | 1.097 |  | .422 | .570 |  | -11.290 | .000 | .441 |
| *Use/Pay* |  |  |  |  |  |  |  |  |  |  |
| Intention |  | -1.059 | .857 |  | .492 | .585 |  | -14.533 | .000 | .566 |
| Pay |  | -.476 | .498 |  | .212 | 1.079 |  | -4.649 | .000 | .117 |

*Note.* Analysis is done based on standardized (Z) scores. Country; Health/Activity = Healthy Lifestyle scale; Motivation-EAT=Motivation to eat healthily; Barrier-EAT = Barriers to eating healthily; Solution -EAT = Solutions to eating healthily; Motivation-PHYSIC= Motivation to do physical activity and exercise; Barrier -PHYSIC = Barriers to physical activity and exercise; Solution -PHYSIC = Solutions for physical activity/exercise; F1= Social support, connectedness and mindfulness; F2= Goal setting, tracking, and advice for exercising; F3= Tips and advice for food and home workouts; F4= Digital score connection and mood management; Health Confidence = Perception of ability and confidence for healthy eating and exercise; Health Maintenance = Perception of ability to maintain healthy eating and exercise habits; Intention = Intention to use the app; Pay = Willingness to pay for the app.

Table S12

*Descriptive statistics of demographic/geographic variables and the questions related to using apps for healthy eating and physical activity in Clusters 1 (Low Health App Users) and 2 (Motivated Health App Enthusiasts)*

| Demographic/Geographic variables |  | Cluster | |
| --- | --- | --- | --- |
|  |  | 1 | 2 |
| Gender |  |  |  |
| Female |  | 24 (41.4%) | 70 (52.6%) |
| Male |  | 33 (56.9%) | 63 (47.4%) |
| Other |  | 1 (1.6%) | 0 (0.0%) |
|  | Total | 58 (100%) | 133 (100%) |
| Education |  |  |  |
| Less than High School |  | 1 (1.7%) | 1 (0.8%) |
| High School / GCSE |  | 2 (3.4%) | 12 (9%) |
| A Levels |  | 10 (17.2%) | 20 (15 %) |
| Bachelors degree |  | 17 (29.3%) | 54 (40.6%) |
| Masters degree |  | 21 (36.2%) | 41 (30.8%) |
| Doctoral Degree |  | 4 (6.9%) | 4 (3 %) |
| Other |  | 3 (5.2%) | 1 (0.8%) |
|  | Total | 58 (100%) | 133 (100%) |
|  |  |  |  |
| Family status |  |  |  |
| Single without child/children |  | 21 (36.2%) | 51 (38.3%) |
| Single with child/children from present or past relationship |  | 1 (1.7%) | 10 (7.5%) |
| Couple without child/children |  | 14 (24.1%) | 39 (29.3%) |
| Couple with child/children from present or past relationship |  | 22 (37.9%) | 33 (24.8%) |
|  | Total | 58 (100%) | 133 (100%) |
|  |  |  |  |
| Country |  |  |  |
| UK |  | 8 (13.8%) | 43 (32.3%) |
| Germany |  | 16 (27.6%) | 36 (27.1%) |
| France |  | 22 (37.9%) | 23 (17.3%) |
| Italy |  | 12 (20.7%) | 31 (22.5%) |
|  | Total | 62 (100%) | 129 (100%) |
|  |  |  |  |
| What do you think about using an app for healthy eating? |  |  |  |
| I have never thought about using an app for that. |  | 39 (67.2%) | 19 (14.3%) |
| I have thought about using an app for that, but so far I did not do it. |  | 8 (13.8%) | 41 (30.8%) |
| I have thought about using an app for that, but it is not necessary for me to do it |  | 3 (5.2%) | 16 (12%) |
| I am currently using an app for that and intend to continue to use it |  | 1 (1.7%) | 19 (14.3%) |
| I have used an app for that, but I do not use it anymore. |  | 7 (12.1%) | 38 (28.6%) |
|  | Total | 58 (100%) | 133 (100%) |
|  |  |  |  |
| What do you think about using an app to for physical activity and exercise? |  |  |  |
| I have never thought about using an app for that. |  | 27 (46.6%) | 9 (6.8%) |
| I have thought about using an app for that, but so far I did not do it. |  | 14 (24.1%) | 38 (28.6%) |
| I have thought about using an app for that, but it is not necessary for me to do it. |  | 3 (5.2%) | 9 (6.8%) |
| I am currently using an app for that and intend to continue to use it. |  | 6 (10.3%) | 34 (25.6%) |
| I have used an app for that, but I do not use it anymore. |  | 8 (13.8%) | 43 (32.3%) |
|  | Total | 58 (100%) | 133 (100%) |

*Figure S2*. App−EAT: using app for healthy eating; App−PA: using app for physical activity; Motivation−EAT: motivation to eat healthily; Barrier−EAT: barriers to eating healthily; Solution−EAT: solutions to eating healthily; Motivation−PHYSIC: motivation to do physical activity and exercise; Barrier−PHYSIC: barriers to physical activity and exercise; Solution−PHYSIC: solutions for physical activity/exercise; F1: social support, connectedness, and mindfulness; F2: goal setting, tracking, and advice for exercising; F3: tips and advice for food and home workouts; F4: digital score connection and mood management.


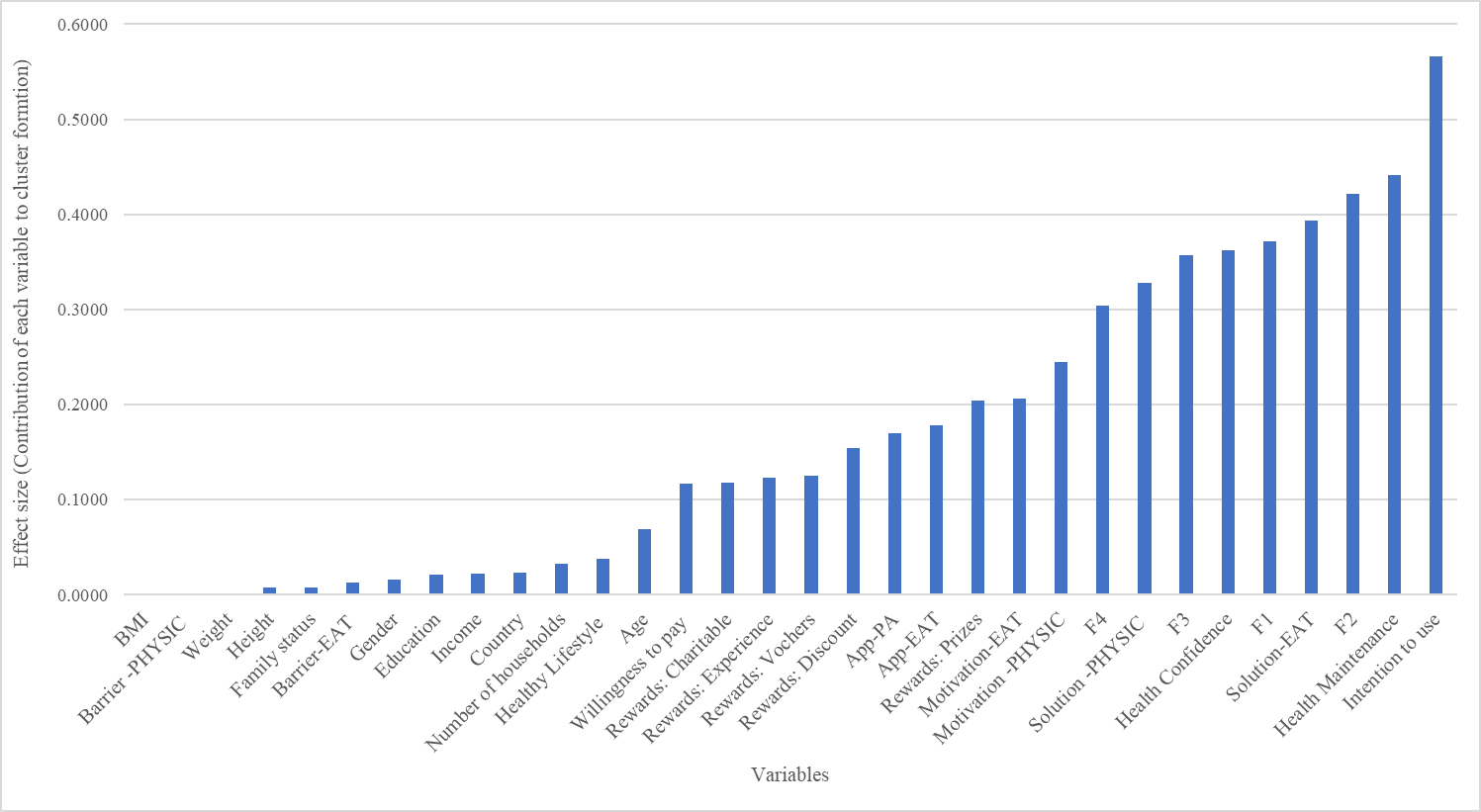


**Backward regression analyses for intention to use an app and willingness to pay for an app**

Table S13

*Summary of backward stepwise regression analysis for variables predicting intention to use the app*

| Predictor |  | *B* | *SE B* | *β* |
| --- | --- | --- | --- | --- |
| Constant |  | -2.950 | .589 |  |
| Digital score connection and mood management |  | .084 | .025 | .153** |
| Health Maintenance |  | .690 | .060 | .574** |
| Cluster Membership |  | 2.222 | .361 | .307** |
| Social support, connectedness and mindfulness × BMI |  | .327 | .116 | .104** |
| Goal setting, tracking, and advice for exercising × Health Confidence |  | .664 | .176 | .319** |
| Tips and advice for food and home workouts × Health Confidence |  | -.722 | .189 | -.316** |
|  |  |  |  |  |
|  |  |  |  |  |
| *R^2^* |  |  | .762 |  |
| Adjusted *R^2^* |  |  | .754 |  |
| *F* |  |  | 98.274*** |  |

*Notes.* F1= Social support, connectedness and mindfulness; F2= Goal setting, tracking, and advice for exercising; F3= Tips and advice for food and home workouts; F4= Digital score connection and mood management; Health Confidence = Perception of ability and confidence for healthy eating and exercise; Health Maintenance = Perception of ability to maintain healthy eating and exercise habits; ** *p* < 0.01; ****p <* .001*.*

**Exploring differences between countries in their attitude towards app features and intention to use.**

Table S14

*One-way ANOVA results to compare the intention to use and attitude towards app factors between countries*

| Variables | df | *F* | Sig |
| --- | --- | --- | --- |
| F1: Social support, connectedness and mindfulness | 3, 206 | 3.158 | . 025 |
| F2: Goal setting, tracking, and advice for exercising | 3, 206 | 1.509 | .213 |
| F3: Tips and advice for food and home workouts | 3, 206 | 3.578 | .015 |
| F4: Digital score connection and mood management | 3, 206 | 5.003 | < .001 |
| Intention to use | 3, 206 | 3.256 | .023 |

**Correlations among variables**

We used correlation analyses (see Table S15) to understand the relationships between consumers’ positive app feature attitudes with their intention to use and willingness to pay for the app and to explore how these variables are associated with differences in consumers' health status, motivational factors, and basic demographics and socioeconomic status (SES).

Table S15

*Correlations between variables*

|  |  | 1 | 2 | 3 | 4 | 5 | 6 | 7 | 8 |
| --- | --- | --- | --- | --- | --- | --- | --- | --- | --- |
| 1-Age | *r* | - |  |  |  |  |  |  |  |
|  | sig |  |  |  |  |  |  |  |  |
| 2-Gender | *r* | .090 | - |  |  |  |  |  |  |
|  | sig | .197 |  |  |  |  |  |  |  |
| 3-Education | *r* | -.041 | .049 | - |  |  |  |  |  |
|  | sig | .554 | .483 |  |  |  |  |  |  |
| 4-Income | *r* | .148* | .049 | .183** | - |  |  |  |  |
|  | sig | .038 | .496 | .010 |  |  |  |  |  |
| 5-BMI | *r* | .175* | .087 | -.218** | -.080 | - |  |  |  |
|  | sig | .013 | .219 | .002 | .270 |  |  |  |  |
| 6-Motivation-EAT | *r* | -.043 | -.075 | -.135 | -.021 | .173* | - |  |  |
|  | sig | .542 | .286 | .052 | .767 | .014 |  |  |  |
| 7-Barrier-EAT | *r* | -.089 | .008 | -.213** | -.125 | .275** | .141* | - |  |
|  | sig | .202 | .909 | .002 | .078 | <.001 | .043 |  |  |
| 8- Solution -EAT | *r* | -.068 | -.151* | -.088 | -.130 | .003 | .626** | .116 | - |
|  | sig | .331 | .030 | .206 | .067 | .971 | <.001 | .096 |  |
| 9-Motivation-PHYSIC | *r* | -.083 | -.057 | -.094 | .059 | .158* | .810** | .163* | .577** |
|  | sig | .237 | .414 | .177 | .411 | .024 | <.001 | .019 | <.001 |
| 10-Barrier-PHYSIC | *r* | -.139* | -.100 | -.186** | -.162* | .187** | .099 | .783** | .097 |
|  | sig | .045 | .153 | .007 | .023 | .008 | .156 | <.001 | .166 |
| 11- Solution -PHYSIC | *r* | -.111 | -.102 | -.066 | -.072 | .065 | .509** | .053 | .707** |
|  | sig | .111 | .144 | .347 | .315 | .358 | <.001 | .449 | <.001 |
| 12-F1 | *r* | -.020 | -.037 | .022 | -.027 | .043 | .423** | .064 | .616** |
|  | sig | .779 | .596 | .751 | .704 | .539 | <.001 | .360 | <.001 |
| 13-F2 | *r* | -.090 | -.113 | -.083 | .065 | .023 | .410** | .058 | .548** |
|  | sig | .197 | .104 | .233 | .364 | .746 | <.001 | .408 | <.001 |
| 14-F3 | *r* | -.152* | -.052 | -.104 | -.126 | .007 | .397** | -.021 | .498** |
|  | sig | .029 | .456 | .137 | .077 | .923 | <.001 | .767 | <.001 |
| 15-F4 | *r* | -.063 | -.060 | -.134 | -.097 | .136 | .421** | .194** | .459** |
|  | sig | .370 | .388 | .055 | .173 | .052 | <.001 | .005 | <.001 |
| 16- Health Confidence | *r* | -.080 | -.020 | -.043 | .055 | .052 | .424** | .007 | .488** |
|  | sig | .250 | .775 | .537 | .440 | .460 | <.001 | .922 | <.001 |
| 17- Health Maintenance | *r* | -.118 | -.073 | -.100 | .007 | .106 | .446** | .016 | .548** |
|  | sig | .091 | .297 | .151 | .923 | .131 | <.001 | .816 | <.001 |
| 18-Intention | *r* | -.105 | -.021 | -.086 | .005 | .078 | .469** | .068 | .564** |
|  | sig | .133 | .761 | .221 | .940 | .271 | <.001 | .330 | <.001 |
| 19-Pay | *r* | -.088 | -.020 | .040 | .053 | .075 | .156* | .029 | .233** |
|  | sig | .211 | .773 | .572 | .465 | .291 | .026 | .679 | <.001 |
| 20-Health/Activity | *r* | .093 | -.055 | .132 | .146* | -.264** | .268** | -.477** | .241** |
|  | sig | .182 | .433 | .059 | .039 | <.001 | <.001 | <.001 | <.001 |
| 21-Cluster Membership | *r* | -.244** | -.117 | -.136 | -.139 | .017 | .427** | .107 | .597** |
|  | sig | <.001 | .107 | .061 | .055 | .819 | <.001 | .141 | <.001 |

*Notes.* F1= Social support, connectedness and mindfulness; F2= Goal setting, tracking, and advice for exercising; F3= Tips and advice for food and home workouts; F4= Digital score connection and mood management; Health Confidence = Perception of ability and confidence for healthy eating and exercise; Health Maintenance = Perception of ability to maintain healthy eating and exercise habits; Intention = Intention to use the app; Pay = Willingness to pay for the app; * *p* < 0.05; ** *p* < 0.01.

Table S15 Continuance

*Correlations between variables*

|  |  | 9 | 10 | 11 | 12 | 13 | 14 | 15 | 16 | 17 | 18 | 19 | 20 | 21 |
| --- | --- | --- | --- | --- | --- | --- | --- | --- | --- | --- | --- | --- | --- | --- |
| 9- Motivation -PHYSIC | *r* | 1 |  |  |  |  |  |  |  |  |  |  |  |  |
|  | sig |  |  |  |  |  |  |  |  |  |  |  |  |  |
| 10-Barrier-PHYSIC | *r* | .069 | 1 |  |  |  |  |  |  |  |  |  |  |  |
|  | sig | .321 |  |  |  |  |  |  |  |  |  |  |  |  |
| 11- Solution -PHYSIC | *r* | .570** | .020 | 1 |  |  |  |  |  |  |  |  |  |  |
|  | sig | <.001 | .770 |  |  |  |  |  |  |  |  |  |  |  |
| 12-F1 | *r* | .425** | .043 | .573** | 1 |  |  |  |  |  |  |  |  |  |
|  | sig | <.001 | .538 | <.001 |  |  |  |  |  |  |  |  |  |  |
| 13-F2 | *r* | .435** | .024 | .475** | .626** | 1 |  |  |  |  |  |  |  |  |
|  | sig | <.001 | .728 | <.001 | <.001 |  |  |  |  |  |  |  |  |  |
| 14-F3 | *r* | .329** | -.024 | .393** | .546** | .653** | 1 |  |  |  |  |  |  |  |
|  | sig | <.001 | .732 | <.001 | <.001 | <.001 |  |  |  |  |  |  |  |  |
| 15-F4 | *r* | .399** | .226** | .342** | .515** | .530** | .494** | 1 |  |  |  |  |  |  |
|  | sig | <.001 | .001 | <.001 | <.001 | <.001 | <.001 |  |  |  |  |  |  |  |
| 16- Health Confidence | *r* | .447** | -.045 | .385** | .476** | .633** | .433** | .406** | 1 |  |  |  |  |  |
|  | sig | <.001 | .525 | <.001 | <.001 | <.001 | <.001 | <.001 |  |  |  |  |  |  |
| 17- Health Maintenance | *r* | .515** | -.037 | .481** | .528** | .678** | .531** | .427** | .800** | 1 |  |  |  |  |
|  | sig | <.001 | .594 | <.001 | <.001 | <.001 | <.001 | <.001 | <.001 |  |  |  |  |  |
| 18-Intention | *r* | .530** | .029 | .533** | .546** | .612** | .467** | .493** | .740** | .813** | - |  |  |  |
|  | sig | <.001 | .683 | <.001 | <.001 | <.001 | <.001 | <.001 | <.001 | <.001 |  |  |  |  |
| 19-Pay | *r* | .204** | .082 | .209** | .312** | .215** | .151* | .196** | .272** | .338** | .319** | - |  |  |
|  | sig | .003 | .242 | .003 | <.001 | .002 | .032 | .005 | <.001 | <.001 | <.001 |  |  |  |
| 20-Health / Activity | *r* | .235** | -.527** | .289** | .242** | .167* | .123 | -.034 | .321** | .239** | .237** | .058 | - |  |
|  | sig | <.001 | <.001 | <.001 | <.001 | .016 | .078 | .628 | <.001 | <.001 | <.001 | .410 |  |  |
| 21- Cluster Membership | *r* | .467** | .023 | .551** | .581** | .620** | .569** | .522** | .572** | .635** | .726** | .320** | .224** | - |
|  | sig | <.001 | .748 | <.001 | <.001 | <.001 | <.001 | <.001 | <.001 | <.001 | <.001 | <.001 | .002 |  |

*Notes.* F1= Social support, connectedness and mindfulness; F2= Goal setting, tracking, and advice for exercising; F3= Tips and advice for food and home workouts; F4= Digital score connection and mood management; Health Confidence = Perception of ability and confidence for healthy eating and exercise; Health Maintenance = Perception of ability to maintain healthy eating and exercise habits; Intention = Intention to use the app; Pay = Willingness to pay for the app; * *p* < 0.05; ** *p* < 0.01.

**Predicting roles of factors of app feature attitudes on self-efficacy**

To investigate whether factors of app feature attitudes predict ‘*perception of ability and confidence for healthy eating and exercise’* and ‘*perception of ability to maintain healthy eating and exercise habits’*, we used two multiple regression analyses (see Table S16 and S17).

Table S16

*Regression analysis summary for factors of app feature attitudes predicting perception of ability and confidence for healthy eating and exercise*

|  | *B* | *SE* | *β* | Sig |
| --- | --- | --- | --- | --- |
| (Constant) | 3.099 | 1.575 |  | .051 |
| F1: Social support, connectedness and mindfulness | .050 | .032 | .114 | .119 |
| F2: Goal setting, tracking, and advice for exercising | .245 | .037 | .529* | <.001 |
| F3: Tips and advice for food and home workouts | -.0005 | .043 | -.009 | .900 |
| F4: Digital score connection and mood management | .064 | .058 | .073 | .276 |
|  |  |  |  |  |
| *R^2^* |  |  | .415 |  |
| Adjusted *R^2^* |  |  | .403 |  |
| *F* |  |  | 35.677 | <.001 |

Multiple regression analysis is used to test which one of the factors related to app features significantly predict participants' ‘*perception of ability and confidence for healthy eating and exercise’* (see Table S16). The results of the regression indicate that the only factor two (‘*goal setting, tracking, and advice for exercising’*) significantly predicts participants’ ‘*perception of ability and confidence for healthy eating and exercise’* (*β* = .529, *p* < .001).

Table S17

*Regression analysis summary for factors of app features predicting perception of ability to maintain healthy eating and exercise habits*

|  | *B* | *SE* | *β* | Sig |
| --- | --- | --- | --- | --- |
| (Constant) | -.078 | .797 |  | .922 |
| F1: Social support, connectedness and mindfulness | .031 | .016 | .134 | .051 |
| F2: Goal setting, tracking, and advice for exercising | .125 | .019 | .501*** | <.001 |
| F3: Tips and advice for food and home workouts | .035 | .022 | .112 | .109 |
| F4: Digital score connection and mood management | .017 | .029 | .037 | .560 |
|  |  |  |  |  |
| *R^2^* |  |  | .486 |  |
| Adjusted *R^2^* |  |  | .475 |  |
| *F* |  |  | 47.666 | <.001 |

Multiple regression analysis is used to test which one of the factors related to app features significantly predict participants' ‘*perception of ability to maintain healthy eating and exercise habits’* (see Table S17). The results of the regression indicate that the only factor two (‘*goal setting, tracking, and advice for exercising’*) significantly predicts participant’s ‘*perception of ability to maintain healthy eating and exercise habits’* (*β* = .501, *p* < .001).

**Mediating impact of self-efficacy**

We used Process Macro model 4 to investigate whether self-efficacy (‘*perception of ability and confidence for healthy eating and exercise’* and ‘*perception of ability to maintain healthy eating and exercise habits’*) mediates the relationship between intention to use and intention to pay for the app.

*Figure S3*. Direct and indirect effects of intention to use the app on willingness to pay for the app through perception of ability to maintain healthy eating and exercise habits; Health Maintenance = Perception of ability to maintain healthy eating and exercise habits; * *p* < 0.05; ** *p* < 0.01*.*

Health Maintenance

Intention to use app

Willing to pay

.40⁕

.66⁕⁕

(.18 [n.s])

.45⁕⁕

The results indicate a significant mediating impact of ‘*perception of ability to maintain healthy eating and exercise habits’* on the link between intention to use the app and willingness to pay for the app, B =.26, *SE* = .12, CI [.04, .53]. This means that higher intention to use the app is associated with higher ‘*perception of ability to maintain healthy eating and exercise habits’* which in turn leads to higher willingness to pay for the app.

*Figure S4*. Direct and indirect effects of intention to use the app on willingness to pay for the app through perception of ability and confidence for healthy eating and exercise; Health Confidence = Perception of ability and confidence for healthy eating and exercise; * *p* < 0.05; ** *p* < 0.01*.*

Health Confidence

Intention to use app

Willing to pay

.07 (n.s)

1.12⁕⁕

(.38⁕⁕)

.46⁕⁕

However, the results do not indicate a significant mediating impact of ‘*perception of ability and confidence for healthy eating and exercise’* on the link between intention to use app and willingness to pay for the app, B= .08, *SE* = .11, CI [-.14, .30].

Table S18

*Summary of backward stepwise regression analysis for variables predicting intention to use the app*

|  | Step **1** | Step **2** | Step **3** | Step **4** | Step **5** | Step **6** | Step **7** | Step **8** | Step **9** | Step **10** |
| --- | --- | --- | --- | --- | --- | --- | --- | --- | --- | --- |
| Variables | *b* | *b* | *b* | *b* | *b* | *b* | *b* | *b* | *b* | *b* |
| F1 | .148* | .148* | .147* | .145* | .145* | .145* | .144* | .144* | .138* | .136* |
| F2 | -.035 | -.035 | -.035 | -.034 | -.035 | -.036 | -.032 | -.033 | -.043 | -.047 |
| F3 | -.030 | -.030 | -.029 | -.029 | -.029 | -.026 | -.028 | -.026 |  |  |
| F4 | .179** | .178** | .178** | .178** | .177** | .177** | .177** | .178** | .176** | .181** |
| BMI | -.086 | -.086 | -.087 | -.088 | -.086 | -.084 | -.085 | -.085 | -.085 | -.083 |
| Health Confidence | .206** | .206** | .205** | .205** | .204** | .204** | .204** | .200** | .203** | .198** |
| Health Maintenance | .534** | .534** | .535** | .537** | .538** | .537** | .537** | .539** | .535** | .542** |
| F1 × BMI | .104 | .102 | .102 | .105 | .109 | .102 | .102 | .104 | .107* | .110* |
| F1 × Health Confidence | -.019 | -.019 | -.019 |  |  |  |  |  |  |  |
| F1 × Health Maintenance | -.088 | -.088 | -.088 | -.103 | -.107 | -.103 | -.102 | -.102 | -.103 | -.104 |
| F2 × BMI | -.005 |  |  |  |  |  |  |  |  |  |
| F2 × Health Confidence | .449** | .448** | .450** | .449** | .445** | .447** | .452** | .458** | .453** | .404** |
| F2 × Health Maintenance | -.080 | -.080 | -.080 | -.081 | -.077 | -.078 | -.081 | -.080 | -.066 |  |
| F3 × BMI | -.093 | -.095 | -.095 | -.096 | -.094 | -.094 | -.094 | -.094 | -.096 | -.099 |
| F3 × Health Confidence | -.206 | -.205 | -.205 | -.213 | -.214 | -.212 | -.214 | -.225 | -.223 | -.210 |
| F3 × Health Maintenance | -.094 | -.094 | -.094 | -.087 | -.084 | -.089 | -.090 | -.084 | -.089 | -.114 |
| F4 × BMI | .111 | .112 | .112* | .112* | .112* | .111* | .109 | .108 | .106 | .103 |
| F4 × Health Confidence | -.065 | -.064 | -.064 | -.070 | -.079 | -.087 | -.091 | -.088 | -.090 | -.076 |
| F4 × Health Maintenance | .069 | .068 | .066 | .073 | .085 | .088 | .093 | .090 | .086 | .069 |
| BMI × Health Confidence | -.025 | -.027 | -.028 | -.029 | -.014 |  |  |  |  |  |
| BMI× Health Maintenance | .023 | .023 | .024 | .023 |  |  |  |  |  |  |
| Age | -.025 | -.024 | -.024 | -.024 | -.024 | -.024 | -.024 | -.028 | -.026 | -.024 |
| Gender | .027 | .027 | .027 | .027 | .027 | .027 | .028 | .030 | .029 | .028 |
| Number of households | -.019 | -.020 | -.019 | -.020 | -.019 | -.019 | -.016 | -.022 | -.022 | -.024 |
| Family status | -.019 | -.018 | -.019 | -.019 | -.018 | -.019 | -.015 |  |  |  |
| Education | -.047 | -.048 | -.047 | -.047 | -.045 | -.044 | -.042 | -.042 | -.041 | -.041 |
| Income | .015 | .015 | .014 | .014 | .012 | .012 |  |  |  |  |
| Country | .005 | .005 |  |  |  |  |  |  |  |  |

*Notes.* F1= Social support, connectedness and mindfulness; F2= Goal setting, tracking, and advice for exercising; F3= Tips and advice for food and home workouts; F4= Digital score connection and mood management; Health Confidence = Perception of ability and confidence for healthy eating and exercise; Health Maintenance = Perception of ability to maintain healthy eating and exercise habits; Intention = Intention to use the app; Pay = Willingness to pay for the app; * *p* < 0.05; ** *p* < 0.01.

Table S18 Continuance

|  | Step **11** | Step **12** | Step **13** | Step **14** | Step **15** | Step **16** | Step **17** | Step **18** | Step **19** | Step **20** | Step **21** | Step **22** | Step **23** |
| --- | --- | --- | --- | --- | --- | --- | --- | --- | --- | --- | --- | --- | --- |
| Variables | *b* | *b* | *b* | *b* | *b* | *b* | *b* | *b* | *b* | *b* | *b* | *b* | *b* |
| F1 | .136* | .134* | .133* | .130* | .132* | .132* | .117* | .110* | .112* | .111* | .101 | .082 |  |
| F2 | -.047 | -.049 | -.048 | -.050 | -.057 | -.049 |  |  |  |  |  |  |  |
| F3 |  |  |  |  |  |  |  |  |  |  |  |  |  |
| F4 | .185** | .192 | .193** | .196** | .198** | .195** | .185** | .191** | .177** | .175** | .189** | .188** | .219** |
| BMI | -.081 | -.082 | -.081 | -.087* | -.084 | -.083 | -.079 | -.071 |  |  |  |  |  |
| Health Confidence | .196** | .200** | .200** | .198** | .203** | .183** | .178** | .177** | .191** | .193** | .186** | .181** | .182** |
| Health Maintenance | .543** | .535** | .536** | .541** | .534** | .558** | .545** | .550** | .539** | .543** | .547** | .573** | .602** |
| F1 × BMI | .109* | .114* | .111* | .111* | .109* | .106* | .109* | .105* | .109* | .084 | .108** | .102* | .114** |
| F1 × Health Confidence |  |  |  |  |  |  |  |  |  |  |  |  |  |
| F1 × Health Maintenance | -.106 | -.098 | -.100 | -.099 | -.100 | -.116* | -.114 | -.113 | -.113 | -.116* | -.108 |  |  |
| F2 × BMI |  |  |  |  |  |  |  |  |  |  |  |  |  |
| F2 × Health Confidence | .402 | .400** | .391** | .387** | .389** | .389** | .391** | .386** | .376** | .370** | .392** | .314** | .315** |
| F2 × Health Maintenance |  |  |  |  |  |  |  |  |  |  |  |  |  |
| F3 × BMI | -.096 | -.097 | -.097 | -.099 | -.094 | -.092 | -.095 | -.093 | -.070 |  |  |  |  |
| F3 × Health Confidence | -.217 | -.246* | -.248* | -.250* | -.247* | -.304** | -.297** | -.291** | -.276** | -.270** | -.294** | -.289** | -.294** |
| F3 × Health Maintenance | -.105 | -.075 | -.071 | -.071 | -.082 |  |  |  |  |  |  |  |  |
| F4 × BMI | .100 | .104 | .103 | .105 | .102 | .100 | .100 | .099 | .076 | .047 |  |  |  |
| F4 × Health Confidence | -.075 | -.019 |  |  |  |  |  |  |  |  |  |  |  |
| F4 × Health Maintenance | .068 |  |  |  |  |  |  |  |  |  |  |  |  |
| BMI × Health Confidence |  |  |  |  |  |  |  |  |  |  |  |  |  |
| BMI × Health Maintenance |  |  |  |  |  |  |  |  |  |  |  |  |  |
| Age | -.028 | -.029 | -.029 | - |  |  |  |  |  |  |  |  |  |
| Gender | .027 | .029 | .030 | .027 |  |  |  |  |  |  |  |  |  |
| Number of households |  |  |  |  |  |  |  |  |  |  |  |  |  |
| Family status |  |  |  |  |  |  |  |  |  |  |  |  |  |
| Education | -.040 | -.041 | -.041 | -.039 | -.038 | -.038 | -.035 |  |  |  |  |  |  |
| Income |  |  |  |  |  |  |  |  |  |  |  |  |  |
| Country |  |  |  |  |  |  |  |  |  |  |  |  |  |

*Notes.* F1= Social support, connectedness and mindfulness; F2= Goal setting, tracking, and advice for exercising; F3= Tips and advice for food and home workouts; F4= Digital score connection and mood management; Health Confidence = Perception of ability and confidence for healthy eating and exercise; Health Maintenance = Perception of ability to maintain healthy eating and exercise habits; Intention = Intention to use the app; * *p* < 0.05; ** *p* < 0.01

Table S19

*Summary of backward stepwise regression analysis for variables predicting* *willingness to pay for the app*

|  | Step1 | Step 2 | Step 3 | Step 4 | Step 5 | Step 6 | Step 7 | Step 8 | Step 9 | Step 10 | Step 11 | Step 12 | Step 13 | Step 14 | Step 15 | Step 16 |
| --- | --- | --- | --- | --- | --- | --- | --- | --- | --- | --- | --- | --- | --- | --- | --- | --- |
| Variables | *b* | *b* | *b* | *b* | *b* | *b* | *b* | *b* | *b* | *b* | *b* | *b* | *b* | *b* | *b* | *b* |
| F1 | .252* | .251* | .251* | .251* | .255* | .254* | .252* | .255* | .255* | .258* | .247* | .245* | .256* | .258* | .259* | .265* |
| F2 | -.048 | -.048 | -.048 | -.051 | -.051 | -.053 | -.047 | -.048 | -.046 | -.045 |  |  |  |  |  |  |
| F3 | -.090 | -.090 | -.091 | -.090 | -.091 | -.092 | -.094 | -.095 | -.097 | -.097 | -.112 | -.114 | -.107 | -.108 | -.104 | -.107 |
| F4 | .055 | .055 | .055 | .056 | .054 | .053 | .052 | .046 | .046 | .041 | .035 | .038 |  |  |  |  |
| BMI | .034 | .034 | .034 | .035 | .030 | .031 | .029 | .032 | .034 | .035 | .037 | .035 | .040 | .038 | .038 | .040 |
| Able | .039 | .039 | .038 | .040 | .042 | .045 | .046 | .052 | .051 | .053 | .044 |  |  |  |  |  |
| Health Maintenance | .176 | .177 | .178 | .175* | .174 | .173 | .173 | .172 | .177 | .170 | .163 | .194 | .191 | .192 | .193 | .189 |
| F1 × BMI | .051 | .052 | .051 | .050 | .047 | .045 | .046 | .036 | .026 |  |  |  |  |  |  |  |
| F1 × Health Confidence | -.006 |  |  |  |  |  |  |  |  |  |  |  |  |  |  |  |
| F1 × Health Maintenance | .113 | .109 | .107 | .106 | .103 | .116 | .113 | .116 | .103 | .109 | .101 | .098 | .107 | .123 | .123 | .188 |
| F2 × BMI | -.048 | -.048 | -.044 | -.044 | -.042 | -.046 | -.048 | -.046 |  |  |  |  |  |  |  |  |
| F2 × Health Confidence | .247 | .248 | .245 | .239 | .241 | .217 | .225 | .226 | .228 | .228 | .222 | .198 | .192 | .271 | .311 | .292 |
| F2 × Health Maintenance | .107 | .105 | .110 | .115 | .116 | .164 | .161 | .158 | .147 | .149 | .144 | .161 | .142 |  |  |  |
| F3 × BMI | -.127 | -.127 | -.129 | -.129 | -.136 | -.133 | -.133 | -.136 | -.152 | -.144 | -.144 | -.145 | -.141 | -.139 | -.134 | -.133 |
| F3 × Health Confidence | -.240 | -.243 | -.241 | -.235 | -.232 | -.201 | -.207 | -.208 | -.205 | -.201 | -.183 | -.181 | -.164 | -.169 | -.225 | -.228 |
| F3 × Health Maintenance | .070 | .072 | .069 | .063 | .066 |  |  |  |  |  |  |  |  |  |  |  |
| F4 × BMI | -.016 | -.016 | -.015 | -.017 |  |  |  |  |  |  |  |  |  |  |  |  |
| F4 × Health Confidence | -.253 | -.254 | -.251 | -.252 | -.252 | -.253 | -.263 | -.257 | -.251 | -.258 | -.253 | -.250 | -.269 | -.282 | -.279 | -.272 |
| F4 × Health Maintenance | .145 | .147 | .145 | .144 | .141 | .132 | .147 | .142 | .151 | .152 | .157 | .158 | .172 | .203 | .193 | .152 |
| BMI × Health Confidence | -.176 | -.176 | -.179 | -.180 | -.180 | -.177 | -.173 | -.188 | -.206 | -.202 | -.207 | -.201 | -.199 | -.194 | -.196 | -.197 |
| BMI × Health Maintenance | .264 | .263 | .265 | .266 | .272 | .272 | .270 | .244 | .244 | .257 | .260 | .254 | .245 | .241 | .243 | .240 |
| Intention | .145 | .144 | .144 | .145 | .144 | .143 | .143 | .136 | .132 | .138 | .142 | .150 | .163 | .158 | .157 | .156 |
| F1 × Intention | .081 | .080 | .084 | .085 | .087 | .077 | .082 | .079 | .094 | .094 | .105 | .106 | .092 | .077 | .072 |  |
| F2 × Intention | -.217 | -.216 | -.217 | -.216 | -.224 | -.241 | -.241 | -.245 | -.246 | -.254 | -.247 | -.242 | -.238 | -.187 | -.228 | -.208 |
| F3 × Intention | -.106 | -.106 | -.105 | -.108 | -.109 | -.083 | -.084 | -.075 | -.071 | -.069 | -.076 | -.078 | -.071 | -.067 |  |  |
| F4 × Intention | .109 | .109 | .106 | .107 | .111 | .119 | .115 | .119 | .100 | .105 | .099 | .096 | .101 | .086 | .090 | .128 |
| BMI × Intention | -.043 | -.043 | -.042 | -.041 | -.051 | -.048 | -.050 |  |  |  |  |  |  |  |  |  |
| Health Confidence × Intention | .221 | .220 | .221 | .222 | .224 | .216 | .220 | .221 | .221 | .226 | .216 | .206 | .208 | .178 | .185 | .192 |
| Health Maintenance × Intention | -.182 | -.181 | -.183 | -.183 | -.185 | -.179 | -.184 | -.187 | -.188 | -.198 | -.194 | -.182 | -.179 | -.140 | -.147 | -.154 |
| Age | -.079 | -.079 | -.079 | -.077 | -.076 | -.076 | -.074 | -.073 | -.072 | -.073 | -.073 | -.072 | -.072 | -.074 | -.080 | -.078 |
| Gender | .010 | .010 | .010 |  |  |  |  |  |  |  |  |  |  |  |  |  |
| Number of households | .008 | .008 |  |  |  |  |  |  |  |  |  |  |  |  |  |  |
| Family status | .050 | .050 | .053 | .051 | .049 | .047 | .055 | .057 | .058 | .057 | .055 | .057 | .056 | .054 | .059 | .061 |
| Education | .038 | .038 | .038 | .038 | .038 | .037 | .042 | .039 | .038 | .040 | .040 | .041 | .039 | .040 | .039 | .038 |
| Income | .020 | .020 | .022 | .023 | .024 | .024 |  |  |  |  |  |  |  |  |  |  |
| Country | .079 | .079 | .080 | .081 | .080 | .080 | .077 | .080 | .081 | .080 | .080 | .079 | .077 | .076 | .077 | .077 |

*Notes.* F1= Social support, connectedness and mindfulness; F2= Goal setting, tracking, and advice for exercising; F3= Tips and advice for food and home workouts; F4= Digital score connection and mood management; Health Confidence = Perception of ability and confidence for healthy eating and exercise; Health Maintenance = Perception of ability to maintain healthy eating and exercise habits; Intention = Intention to use the app; * *p* < 0.05; ** *p* < 0.01

Table S19 Continuance

|  | Step 17 | Step 18 | Step 19 | Step 20 | Step 21 | Step 22 | Step 23 | Step 24 | Step 25 | Step 26 | Step 27 | Step 28 | Step 29 | Step 30 | Step 31 | Step 32 | Step 33 | Step 34 | Step 35 |
| --- | --- | --- | --- | --- | --- | --- | --- | --- | --- | --- | --- | --- | --- | --- | --- | --- | --- | --- | --- |
| Variables | *b* | *b* | *b* | *b* | *b* | *b* | *b* | *b* | *b* | *b* | *b* | *b* | *b* | *b* | *b* | *b* | *b* | *b* | *b* |
| F1 | .274* | .273* | .271* | .264* | .260* | .247* | .246* | .238* | .205* | .194* | .191* | .196* | .176* | .182* | .212* | .207* | .210* | .207* | .229** |
| F2 |  |  |  |  |  |  |  |  |  |  |  |  |  |  |  |  |  |  |  |
| F3 | -.116 | -.114 | -.114 | -.113 | -.110 | -.095 | -.103 | -.099 |  |  |  |  |  |  |  |  |  |  |  |
| F4 |  |  |  |  |  |  |  |  |  |  |  |  |  |  |  |  |  |  |  |
| BMI | .033 |  |  |  |  |  |  |  |  |  |  |  |  |  |  |  |  |  |  |
| Health Confidence |  |  |  |  |  |  |  |  |  |  |  |  |  |  |  |  |  |  |  |
| Health Maintenance | .194 | .195 | .175 | .208 | .201 | .206 | .217 | .218 | .191 | .226 | .226 | .214 | .192 | .199 | .319* | .321* | .302* | .305* | .237** |
| F1 × BMI |  |  |  |  |  |  |  |  |  |  |  |  |  |  |  |  |  |  |  |
| F1 × Health Confidence |  |  |  |  |  |  |  |  |  |  |  |  |  |  |  |  |  |  |  |
| F1 × Health Maintenance | .188 | .191 | .209 | .193 | .203 | .199 | .197 | .201 | .209* | .215* | .201 | .179 | .220 | .159* | .154* | .132 | .136 | .136 |  |
| F2 × BMI |  |  |  |  |  |  |  |  |  |  |  |  |  |  |  |  |  |  |  |
| F2 × Health Confidence | .308 | .324 | .341 | .372 | .406 | .422 | .399 | .385 | .440 | .305 | .255 | .168 |  |  |  |  |  |  |  |
| F2 × Health Maintenance |  |  |  |  |  |  |  |  |  |  |  |  |  |  |  |  |  |  |  |
| F3 × BMI | -.133 | -.139 | -.140 | -.140 | -.135 | -.133 | -.134 | -.133 | -.154 | -.151* | -.146 | -.139 | -.132 | -.124 | -.120 |  |  |  |  |
| F3 × Health Confidence | -.235 | -.244 | -.252 | -.245 | -.244 | -.247 | -.235 | -.232 | -.259* | -.235 | -.223 | -.218 | -.100 |  |  |  |  |  |  |
| F3 × Health Maintenance |  |  |  |  |  |  |  |  |  |  |  |  |  |  |  |  |  |  |  |
| F4 × BMI |  |  |  |  |  |  |  |  |  |  |  |  |  |  |  |  |  |  |  |
| F4 × Health Confidence | -.261 | -.261 | -.185 | -.184 | -.179 | -.182 | -.178 | -.176 | -.206 | -.078 |  |  |  |  |  |  |  |  |  |
| F4 × Health Maintenance | .146 | .131 |  |  |  |  |  |  |  |  |  |  |  |  |  |  |  |  |  |
| BMI × Health Confidence | -.206 | -.221 | -.235 | -.209 | -.202 | -.206 | -.206 | -.211 | -.192 | -.227 | -.253* | -.232 | -.225 | -.243* | -.246* | -.240 | -.086 |  |  |
| BMI × Health Maintenance | .251 | .265 | .287 | .251 | .233 | .234 | .234 | .236 | .247 | .286 | .283* | .247* | .232 | .233 | .244* | .186 |  |  |  |
| Intention | .148 | .149 | .169 | .158 | .155 | .148 | .146 | .152 | .148 | .114 | .122 | .143 | .166 | .168 |  |  |  |  |  |
| F1 × Intention |  |  |  |  |  |  |  |  |  |  |  |  |  |  |  |  |  |  |  |
| F2 × Intention | -.223 | -.231 | -.244 | -.281 | -.269 | -.263 | -.251 | -.251 | -.256 | -.136 | -.121 |  |  |  |  |  |  |  |  |
| F3 × Intention |  |  |  |  |  |  |  |  |  |  |  |  |  |  |  |  |  |  |  |
| F4 × Intention | .127 | .138 | .179 | .184 | .182 | .170 | .167 | .163 | .165 |  |  |  |  |  |  |  |  |  |  |
| BMI × Intention |  |  |  |  |  |  |  |  |  |  |  |  |  |  |  |  |  |  |  |
| Health Confidence × Intention | .180 | .184 | .147 | .074 |  |  |  |  |  |  |  |  |  |  |  |  |  |  |  |
| Health Maintenance × Intention | -.141 | -.143 | -.105 |  |  |  |  |  |  |  |  |  |  |  |  |  |  |  |  |
| Age | -.082 | -.076 | -.077 | -.075 | -.074 | -.070 | -.054 |  |  |  |  |  |  |  |  |  |  |  |  |
| Gender |  |  |  |  |  |  |  |  |  |  |  |  |  |  |  |  |  |  |  |
| Number of households |  |  |  |  |  |  |  |  |  |  |  |  |  |  |  |  |  |  |  |
| Family status | .063 | .061 | .060 | .060 | .063 | .053 |  |  |  |  |  |  |  |  |  |  |  |  |  |
| Education |  |  |  |  |  |  |  |  |  |  |  |  |  |  |  |  |  |  |  |
| Income |  |  |  |  |  |  |  |  |  |  |  |  |  |  |  |  |  |  |  |
| Country | .084 | .073 | .066 | .064 | .058 |  |  |  |  |  |  |  |  |  |  |  |  |  |  |

*Notes.* F1= Social support, connectedness and mindfulness; F2= Goal setting, tracking, and advice for exercising; F3= Tips and advice for food and home workouts; F4= Digital score connection and mood management; Health Confidence = Perception of ability and confidence for healthy eating and exercise; Health Maintenance = Perception of ability to maintain healthy eating and exercise habits; Intention = Intention to use the app; * *p* < 0.05; ** *p* < 0.01

Table S20

*Summary of backward stepwise regression analysis for variables predicting intention to use the app*

|  | Step **1** | Step **2** | Step **3** | Step **4** | Step **5** | Step **6** | Step **7** | Step **8** | Step **9** | Step **10** |
| --- | --- | --- | --- | --- | --- | --- | --- | --- | --- | --- |
| Variables | *b* | *b* | *b* | *b* | *b* | *b* | *b* | *b* | *b* | *b* |
| F1 | .095 | .095 | .094 | .095 | .095 | .094 | .095 | .101 | .094 | .093 |
| F2 | -.092 | -.092 | -.092 | -.091 | -.091 | -.093 | -.093 | -.094 | -.092 | -.094 |
| F3 | -.068 | -.068 | -.067 | -.067 | -.067 | -.064 | -.065 | -.065 | -.062 | -.066 |
| F4 | .145** | .145** | .145** | .145** | .145** | .145** | .145** | .145** | .146** | .149** |
| BMI | -.061 | -.062 | -.062 | -.061 | -.061 | -.057 | -.057 | -.054 | -.050 | -.048 |
| Health Confidence | .171 | .171 | .171* | .171* | .170* | .169* | .169* | .168* | .167* | .172* |
| Health Maintenance | .466** | .466** | .466** | .466** | .466** | .466** | .468** | .464** | .465 | .463** |
| F1 × BMI | .085 | .085 | .087 | .087 | .088 | .085 | .090 | .083 | .081 | .082 |
| F1 × Health Confidence | .072 | .072 | .072 | .073 | .073 | .071 | .072 |  |  |  |
| F1 × Health Maintenance | -.116 | -.116 | -.116 | -.117 | -.117 | -.115 | -.120 | -.063 | -.062 | -.057 |
| F2 × BMI | .006 | .007 |  |  |  |  |  |  |  |  |
| F2 × Health Confidence | .622** | .621** | .620** | .623** | .625** | .624** | .619** | .610** | .611** | .540** |
| F2 × Health Maintenance | -.360 | -.358 | -.354 | -.357 | -.358 | -.355 | -.352 | -.336 | -.338 | -.253 |
| F3 × BMI | -.064 | -.065 | -.062 | -.062 | -.062 | -.060 | -.059 | -.054 | -.053 | -.053 |
| F3 × Health Confidence | -.412* | -.411* | -.411** | -.414** | -.417** | -.415** | -.415** | -.376** | -.372** | -.297** |
| F3 × Health Maintenance | .129 | .128 | .126 | .129 | .131 | .128 | .131 | .099 | .097 |  |
| F4 × BMI | .076 | .077 | .076 | .076 | .075 | .074 | .075 | .075 | .073 | .070 |
| F4 × Health Confidence | -.131 | -.130 | -.131 | -.131 | -.132 | -.148 | -.148 | -.120 | -.123 | -.125 |
| F4 × Health Maintenance | .167 | .166 | .167 | .167 | .168 | .183 | .186 | .159 | .161 | .163 |
| BMI × Health Confidence | -.031 | -.032 | -.029 | -.028 | -.028 |  |  |  |  |  |
| BMI × Health Maintenance | .032 | .033 | .033 | .033 | .032 | .009 |  |  |  |  |
| Age | .005 | .005 | .004 |  |  |  |  |  |  |  |
| Gender | .038 | .038 | .038 | .039 | .039 | .039 | .040 | .041 | .041 | .035 |
| Number of households | .003 |  |  |  |  |  |  |  |  |  |
| Family status | -.006 | -.005 | -.005 | -.004 |  |  |  |  |  |  |
| Education | -.026 | -.026 | -.026 | -.027 | -.026 | -.024 | -.024 | -.024 |  |  |
| Income | .034 | .035 | .035 | .035 | .033 | .032 | .032 | .033 | .029 | .026 |
| Country | .047 | .047 | .047 | .047 | .048 | .049 | .049 | .049 | .044 | .045 |
| Cluster membership | .326** | .325** | .325** | .324** | .325** | .324** | .324** | .322** | .324** | .320** |

*Notes.* F1= Social support, connectedness and mindfulness; F2= Goal setting, tracking, and advice for exercising; F3= Tips and advice for food and home workouts; F4= Digital score connection and mood management; Health Confidence = Perception of ability and confidence for healthy eating and exercise; Health Maintenance = Perception of ability to maintain healthy eating and exercise habits; * *p* < 0.05; ** *p* < 0.01

Table S20 Continuance

|  | Step **11** | Step **12** | Step **13** | Step **14** | Step **15** | Step **16** | Step **17** | Step **18** | Step **19** | Step **20** | Step **21** | Step **22** | Step **23** | Step **24** |
| --- | --- | --- | --- | --- | --- | --- | --- | --- | --- | --- | --- | --- | --- | --- |
| Variables | *b* | *b* | *b* | *b* | *b* | *b* | *b* | *b* | *b* | *b* | *b* | *b* | *b* | *b* |
| F1 | .093 | .082 | .077 | .071 | .073 | .072 | .064 | .064 |  |  |  |  |  |  |
| F2 | -.085 | -.084 | -.086 | -.111 | -.120* | -.125* | -.124* | -.116 | -.093 | -.100 | -.100 | -.099 |  |  |
| F3 | -.070 | -.072 | -.057 |  |  |  |  |  |  |  |  |  |  |  |
| F4 | .147^a^ | .148^a^ | .145^a^ | .142^a^ | .144^a^ | .138^a^ | .147^a^ | .135^a^ | .149^a^ | .167^a^ | .169^a^ | .168^a^ | .142^a^ | .153** |
| BMI | -.052 | -.053 | -.065 | -.064 | -.062 | -.051 | -.045 |  |  |  |  |  |  |  |
| Health Confidence | .175** | .178** | .176** | .186** | .191** | .194** | .187** | .192** | .186** | .185** | .185** | .157* | .146* |  |
| Health Maintenance | .465** | .465** | .469** | .459** | .451** | .451** | .457** | .449** | .464** | .464** | .463** | .504** | .478** | .574** |
| F1 × BMI | .083 | .085 | .085 | .093 | .089 | .069 | .086* | .089* | .096* | .101** | .104** | .098** | .096** | .104** |
| F1 × Able |  |  |  |  |  |  |  |  |  |  |  |  |  |  |
| F1 × Health Maintenance | -.056 |  |  |  |  |  |  |  |  |  |  |  |  |  |
| F2 × BMI |  |  |  |  |  |  |  |  |  |  |  |  |  |  |
| F2 × Health Confidence | .555** | .540** | .553** | .575** | .585** | .579** | .584** | .558** | .539** | .447** | .445** | .329** | .331** | .319** |
| F2 × Health Maintenance | -.262 | -.284 | -.278 | -.276 | -.287* | -.294 | -.276 | -.259 | -.238 | -.136 | -.125 |  |  |  |
| F3 × BMI | -.054 | -.055 | -.057 | -.066 | -.059 |  |  |  |  |  |  |  |  |  |
| F3 × Health Confidence | -.303 | -.305** | -.311** | -.323** | -.329** | -.319** | -.337** | -.324** | -.321** | -.331** | -.332** | -.326** | -.305** | -.316** |
| F3 × Health Maintenance |  |  |  |  |  |  |  |  |  |  |  |  |  |  |
| F4 × BMI | .070 | .067 | .068 | .066 | .062 | .036 |  |  |  |  |  |  |  |  |
| F4 × Health Confidence | -.133 | -.122 | -.122 | -.135 | -.137 | -.142 | -.141 | -.134 | -.123 |  |  |  |  |  |
| F4 × Health Maintenance | .171 | .155 | .141 | .142 | .142 | .148 | .148 | .143 | .134 | .013 |  |  |  |  |
| BMI × Health Confidence |  |  |  |  |  |  |  |  |  |  |  |  |  |  |
| BMI × Health Maintenance |  |  |  |  |  |  |  |  |  |  |  |  |  |  |
| Age |  |  |  |  |  |  |  |  |  |  |  |  |  |  |
| Gender | .037 | .039 | .042 | .041 |  |  |  |  |  |  |  |  |  |  |
| Number of households |  |  |  |  |  |  |  |  |  |  |  |  |  |  |
| Family status |  |  |  |  |  |  |  |  |  |  |  |  |  |  |
| Education |  |  |  |  |  |  |  |  |  |  |  |  |  |  |
| Income |  |  |  |  |  |  |  |  |  |  |  |  |  |  |
| Country | .040 | .040 |  |  |  |  |  |  |  |  |  |  |  |  |
| Cluster membership | .313** | .320** | .311** | .300** | .300** | .308** | .311** | .314** | .325** | .319** | .318** | .316** | .296** | .307** |

*Notes.* F1= Social support, connectedness and mindfulness; F2= Goal setting, tracking, and advice for exercising; F3= Tips and advice for food and home workouts; F4= Digital score connection and mood management; Health Confidence = Perception of ability and confidence for healthy eating and exercise; Health Maintenance = Perception of ability to maintain healthy eating and exercise habits; * *p* < 0.05; ** *p* < 0.01

Table S21

*Summary of backward stepwise regression analysis for variables predicting willingness to pay for the app*

|  | Step1 | Step 2 | Step 3 | Step 4 | Step 5 | Step 6 | Step 7 | Step 8 | Step 9 | Step 10 | Step 11 | Step 12 | Step 13 | Step 14 | Step 15 | Step 16 |
| --- | --- | --- | --- | --- | --- | --- | --- | --- | --- | --- | --- | --- | --- | --- | --- | --- |
| Variables | *b* | *b* | *b* | *b* | *b* | *b* | *b* | *b* | *b* | *b* | *b* | *b* | *b* | *b* | *b* | *b* |
| F1 | .232* | .232* | .234* | .235* | .239* | .246* | .243* | .245* | .243* | .245* | .250* | .251* | .252* | .257* | .267** | .271** |
| F2 | -.075 | -.074 | -.074 | -.078 | -.077 | -.084 | -.077 | -.078 | -.077 | -.081 | -.079 | -.079 | -.078 | -.070 | -.073 | -.076 |
| F3 | -.120 | -.120 | -.120 | -.120 | -.121 | -.121 | -.125 | -.124 | -.127 | -.123 | -.124 | -.125 | -.127 | -.124 | -.130 | -.134 |
| F4 | .046 | .046 | .046 | .046 | .044 | .039 | .042 | .042 | .041 | .038 | .030 | .031 | .028 |  |  |  |
| BMI | .040 | .040 | .041 | .042 | .035 | .036 | .034 | .034 | .034 | .035 | .037 | .039 | .042 | .046 | .040 | .041 |
| Health Confidence | .036 | .036 | .036 | .038 | .041 | .045 |  |  |  |  |  |  |  |  |  |  |
| Health Maintenance | .190 | .190 | .188 | .184 | .182 | .178 | .206 | .206 | .051 | .213 | .206 | .210 | .214 | .210 | .214 | .259* |
| F1 × BMI | .052 | .053 | .050 | .047 | .043 | .045 | .049 | .049 | .051 | .042 |  |  |  |  |  |  |
| F1 × Health Confidence | .020 | .022 |  |  |  |  |  |  |  |  |  |  |  |  |  |  |
| F1 × Health Maintenance | .123 | .122 | .137 | .136 | .132 | .193 | .190 | .192 | .199 | .196 | .205 | .203 | .202 | .200 | .199 | .195 |
| F2 × BMI | -.060 | -.059 | -.058 | -.058 | -.055 | -.068 | -.067 | -.056 | -.056 | -.052 | -.037 |  |  |  |  |  |
| F2 × Health Confidence | .329 | .339 | .340 | .336 | .338 | .327 | .310 | .306 | .312 | .344 | .337 | .328 | .322 | .310 | .322 | .337 |
| F2 × Health Maintenance | .019 |  |  |  |  |  |  |  |  |  |  |  |  |  |  |  |
| F3 × BMI | -.108 | -.108 | -.107 | -.106 | -.117 | -.110 | -.112 | -.118 | -.116 | -.112 | -.108 | -.124 | -.129 | -.127 | -.127 | -.126 |
| F3 × Health Confidence | -.310 | -.314 | -.304 | -.297 | -.292 | -.289 | -.291 | -.286 | -.243 | -.285 | -.273 | -.270 | -.267 | -.249 | -.253 | -.263 |
| F3 × Health Maintenance | .108 | .114 | .109 | .104 | .109 | .088 | .101 | .090 |  |  |  |  |  |  |  |  |
| F4 × BMI | -.023 | -.023 | -.023 | -.024 |  |  |  |  |  |  |  |  |  |  |  |  |
| F4 × Health Confidence | -.285 | -.287 | -.281 | -.283 | -.283 | -.285 | -.281 | -.270 | -.278 | -.276 | -.282 | -.273 | -.265 | -.277 | -.265 | -.271 |
| F4 × Health Maintenance | .169 | .174 | .168 | .168 | .163 | .132 | .134 | .125 | .139 | .133 | .134 | .131 | .129 | .141 | .132 | .157 |
| BMI × Health Confidence | -.169 | -.169 | -.169 | -.170 | -.171 | -.164 | -.159 | -.168 | -.163 | -.168 | -.171 | -.188 | -.200 | -.200 | -.208 | -.208 |
| BMI × Health Maintenance | .267 | .266 | .266 | .267 | .277 | .272 | .270 | .273 | .270 | .268 | .279 | .275 | .248 | .243 | .253 | .256 |
| Intention | .062 | .061 | .062 | .064 | .062 | .062 | .071 | .071 | .069 | .066 | .072 | .068 | .062 | .071 | .066 |  |
| F1 × Intention | .062 | .061 | .063 | .064 | .066 |  |  |  |  |  |  |  |  |  |  |  |
| F2 × Intention | -.245 | -.239 | -.239 | -.237 | -.247 | -.236 | -.222 | -.211 | -.213 | -.248 | -.256 | -.253 | -.250 | -.251 | -.264 | -.270 |
| F3 × Intention | -.087 | -.088 | -.090 | -.094 | -.097 | -.083 | -.092 | -.090 | -.054 |  |  |  |  |  |  |  |
| F4 × Intention | .136 | .134 | .134 | .135 | .139 | .175 | .170 | .165 | .158 | .159 | .164 | .155 | .155 | .156 | .155 | .136 |
| BMI* Intention | -.051 | -.051 | -.050 | -.049 | -.063 | -.061 | -.069 | -.064 | -.062 | -.056 | -.044 | -.044 |  |  |  |  |
| Health Confidence × Intention | .212 | .209 | .211 | .213 | .215 | .215 | .205 | .203 | .175 | .181 | .187 | .189 | .186 | .188 | .175 | .173 |
| Health Maintenance × Intention | -.157 | -.153 | -.154 | -.154 | -.157 | -.155 | -.142 | -.145 | -.116 | -.121 | -.136 | -.137 | -.135 | -.135 | -.121 | -.116 |
| Age | -.062 | -.062 | -.062 | -.060 | -.059 | -.058 | -.057 | -.058 | -.059 | -.062 | -.062 | -.060 | -.059 | -.058 | -.062 | -.061 |
| Gender | .012 | .012 | .013 |  |  |  |  |  |  |  |  |  |  |  |  |  |
| Number of households | .027 | .027 | .027 | .029 | .028 | .032 | .031 |  |  |  |  |  |  |  |  |  |
| Family status | .049 | .049 | .049 | .046 | .043 | .042 | .044 | .055 | .051 | .055 | .054 | .056 | .057 | .056 | .056 | .056 |
| Education | .045 | .045 | .046 | .046 | .045 | .045 | .046 | .043 | .042 | .041 | .042 | .040 | .037 | .036 |  |  |
| Income | .033 | .033 | .034 | .035 | .037 | .038 | .038 | .046 | .044 | .045 | .046 | .047 | .047 | .046 | .054 | .055 |
| Country | .100 | .100 | .100 | .101 | .100 | .099 | .098 | .100 | .100 | .102 | .102 | .103 | .105 | .104 | .110 | .112 |
| Cluster membership | .177 | .177 | .177 | .176 | .175 | .176 | .177 | .171 | .171 | .174 | .175 | .173 | .171 | .173 | .171 | .192 |

*Notes.* F1= Social support, connectedness and mindfulness; F2= Goal setting, tracking, and advice for exercising; F3= Tips and advice for food and home workouts; F4= Digital score connection and mood management; Health Confidence = Perception of ability and confidence for healthy eating and exercise; Health Maintenance = Perception of ability to maintain healthy eating and exercise habits; Intention = Intention to use the app; * *p* < 0.05; ** *p* < 0.01

Table S21 Continuance

|  | Step 17 | Step 18 | Step 19 | Step 20 | Step 21 | Step 22 | Step 23 | Step 24 | Step 25 | Step 26 | Step 27 | Step 28 | Step 29 | Step 30 | Step 31 | Step 32 | Step 33 | Step 34 | Step 35 | Step 36 |
| --- | --- | --- | --- | --- | --- | --- | --- | --- | --- | --- | --- | --- | --- | --- | --- | --- | --- | --- | --- | --- |
| Variables | *b* | *b* | *b* | *b* | *b* | *b* | *b* | *b* | *b* | *b* | *b* | *b* | *b* | *b* | *b* | *b* | *b* | *b* | *b* | *b* |
| F1 | .271** | .271** | .256** | .246* | .248** | .244* | .234* | .235* | .224* | .213* | .215* | .227* | .216* | .221* | .230* | .266** | .292** | .295** | .285** | .229** |
| F2 | -.079 | -.067 |  |  |  |  |  |  |  |  |  |  |  |  |  |  |  |  |  |  |
| F3 | -.130 | -.136 | -.158 | -.157 | -.158 | -.155 | -.157 | -.161 | -.137 | -.134 | -.148 | -.147 | -.164 | -.189* | -.181* | -.151 | -.168 | -.167 | -.149 |  |
| F4 |  |  |  |  |  |  |  |  |  |  |  |  |  |  |  |  |  |  |  |  |
| BMI |  |  |  |  |  |  |  |  |  |  |  |  |  |  |  |  |  |  |  |  |
| Health Confidence |  |  |  |  |  |  |  |  |  |  |  |  |  |  |  |  |  |  |  |  |
| Health Maintenance | .263* | .266* | .251* | .275** | .262** | .254* | .256* | .268** | .272** | .291** | .296** | .300** | .298** | .306** | .318** | .366** | .314** | .291** | .288** | .237** |
| F1 × BMI |  |  |  |  |  |  |  |  |  |  |  |  |  |  |  |  |  |  |  |  |
| F1 × Health Confidence |  |  |  |  |  |  |  |  |  |  |  |  |  |  |  |  |  |  |  |  |
| F1 × Health Maintenance | .198 | .198 | .201 | .191 | .210 | .220 | .224* | .219* | .213* | .218* | .210* | .181 | .218* | .191* | .125 | .116 |  |  |  |  |
| F2 × BMI |  |  |  |  |  |  |  |  |  |  |  |  |  |  |  |  |  |  |  |  |
| F2 × Health Confidence | .358 | .370 | .369 | .409 | .417 | .452 | .436 | .417 | .436 | .288 | .257 | .158 |  |  |  |  |  |  |  |  |
| F2 × Health Maintenance |  |  |  |  |  |  |  |  |  |  |  |  |  |  |  |  |  |  |  |  |
| F3 × BMI | -.133 | -.133 | -.133 | -.133 | -.134 | -.129 | -.128 | -.129 | -.127 | -.124* | -.118 | -.110 | -.100 |  |  |  |  |  |  |  |
| F3 × Health Confidence | -.277 | -.290 | -.278 | -.274 | -.285 | -.283 | -.280 | -.266 | -.270 | -.238 | -.230 | -.229 | -.119 | -.109 |  |  |  |  |  |  |
| F3 × Health Maintenance |  |  |  |  |  |  |  |  |  |  |  |  |  |  |  |  |  |  |  |  |
| F4 × BMI |  |  |  |  |  |  |  |  |  |  |  |  |  |  |  |  |  |  |  |  |
| F4 × Health Confidence | -.274 | -.287 | -.271 | -.238 | -.178 | -.173 | -.171 | -.168 | -.171 | -.042 |  |  |  |  |  |  |  |  |  |  |
| F4 × Health Maintenance | .142 | .167 | .157 | .100 |  |  |  |  |  |  |  |  |  |  |  |  |  |  |  |  |
| BMI × Health Confidence | -.225 | -.219 | -.229 | -.207 | -.227 | -.219 | -.224 | -.223 | -.227 | -.259* | -.274* | -.249* | -.245* | -.245* | -.264* | -.257* | -.265* | -.104 |  |  |
| BMI × Health Maintenance | .272 | .267 | .276 | .246 | .276* | .257 | .259 | .259 | .260 | .293* | .291* | .248* | .235 | .188 | .194 | .189 | .195 |  |  |  |
| Intention |  |  |  |  |  |  |  |  |  |  |  |  |  |  |  |  |  |  |  |  |
| F1 × Intention |  |  |  |  |  |  |  |  |  |  |  |  |  |  |  |  |  |  |  |  |
| F2 × Intention | -.281 | -.280 | -.275 | -.321 | -.321 | -.310 | -.309 | -.293 | -.284 | -.156 | -.150 |  |  |  |  |  |  |  |  |  |
| F3 × Intention |  |  |  |  |  |  |  |  |  |  |  |  |  |  |  |  |  |  |  |  |
| F4 × Intention | .149 | .140 | .140 | .164 | .190 | .189 | .188 | .188 | .165 |  |  |  |  |  |  |  |  |  |  |  |
| BMI* Intention |  |  |  |  |  |  |  |  |  |  |  |  |  |  |  |  |  |  |  |  |
| Health Confidence × Intention | .180 | .187 | .179 | .083 | .075 |  |  |  |  |  |  |  |  |  |  |  |  |  |  |  |
| Health Maintenance × Intention | -.120 | -.129 | -.131 |  |  |  |  |  |  |  |  |  |  |  |  |  |  |  |  |  |
| Age | -.054 | -.054 | -.056 | -.054 | -.054 | -.053 |  |  |  |  |  |  |  |  |  |  |  |  |  |  |
| Gender |  |  |  |  |  |  |  |  |  |  |  |  |  |  |  |  |  |  |  |  |
| Number of households |  |  |  |  |  |  |  |  |  |  |  |  |  |  |  |  |  |  |  |  |
| Family status | .056 | .073 | .071 | .070 | .069 | .072 | .057 |  |  |  |  |  |  |  |  |  |  |  |  |  |
| Education |  |  |  |  |  |  |  |  |  |  |  |  |  |  |  |  |  |  |  |  |
| Income | .049 |  |  |  |  |  |  |  |  |  |  |  |  |  |  |  |  |  |  |  |
| Country | .098 | .094 | .094 | .089 | .084 | .078 | .077 | .066 |  |  |  |  |  |  |  |  |  |  |  |  |
| Cluster membership | .190 | .178 | .170 | .171 | .177 | .175 | .189 | .176 | .158 | .137 | .142 | .142 | .146 | .151 | .139 |  |  |  |  |  |

*Notes.* F1= Social support, connectedness and mindfulness; F2= Goal setting, tracking, and advice for exercising; F3= Tips and advice for food and home workouts; F4= Digital score connection and mood management; Health Confidence = Perception of ability and confidence for healthy eating and exercise; Health Maintenance = Perception of ability to maintain healthy eating and exercise habits; Intention = Intention to use the app; * *p* < 0.05; ** *p* < 0.01
